# Temporal signals in dairy cattle slurry and fertilized field soil resistomes and bacterial communities

**DOI:** 10.3389/fmicb.2025.1666851

**Published:** 2026-01-20

**Authors:** Alexander D. Williams, Stephen P. T. Hooton, Elizabeth King, Lisa M. Avery, Rupert L. Hough, Jon L. Hobman, Dov J. Stekel, Andrew L. Neal, Helen M. West

**Affiliations:** 1 School of Biosciences, Sutton Bonington Campus, University of Nottingham, Loughborough, United Kingdom; 2 State Key Laboratory of Emerging Infectious Diseases, University of Hong Kong, Pokfulam, Hong Kong SAR, China; 3 James Hutton Institute, Aberdeen, United Kingdom; 4 Rothamsted Research, Devon, United Kingdom

**Keywords:** ARGs, MAG, *Proteiniphilum*, resistome, slurry, soil

## Abstract

**Introduction:**

Dairy cattle waste is a globally significant source of organic fertilizer which contains a cocktail of microbes and antibiotic resistance genes (ARGs). These ARGs may present a risk to human and animal health, yet there is still limited farm-system-level understanding of how long-term and multiple slurry applications alter field soil resistomes and total microbial communities.

**Methods:**

Using metagenomics, we assessed both immediate and longer-term changes in grassland field soil resistomes and bacterial communities over a year of routine cattle slurry application.

**Results:**

Our findings suggest that soil microbial communities are resilient to bacteria and ARGs introduced via slurry, even after repeated applications. Most slurry-borne ARGs were not enriched in field soil, however, those common in soil, such as rifamycin resistance genes, were consistently elevated relative to field soil with no history of slurry application. We observed transient increases in slurry-associated macrolide-lincosamide-streptogramin ARGs, however, their persistence appeared to be influenced by timing of slurry application. Similar transient effects were shown by the recovery of a high quality, slurry-associated *Proteiniphilum* spp. metagenome assembled genome (MAG).

**Discussion:**

We show that MAGs represent a powerful tool for examining the transfer of slurry-borne microorganisms, as they can be more characteristic of these environments than typical sentinel organisms which are easily cultivated. Our findings indicate that while the soil bacterial community shows considerable resilience to slurry-borne bacteria and ARGs, this may be diminished by temporal factors that remain largely unexplored and poorly understood. This is important because resilience inferred from short-term observations may not fully capture delayed or transient responses, potentially leading to underestimation of the persistence of slurry-borne bacteria and ARGs.

## 1 Introduction

The emergence and spread of antimicrobial resistance (AMR) continue to cause global concern. Livestock waste is valuable as a means of fertilizing agricultural land worldwide, yet it is also a route by which antibiotic resistant bacteria or genes may be introduced or otherwise enriched in the environment (Larsson and Flach, 2022; Wu et al., 2023). The antibiotic resistome of animal waste, which comprises the pool of antibiotic resistant genes (ARGs) contained within, is shaped by many factors, including the gut microbiome of the animals (Liu et al., 2019; Fu et al., 2023), antibiotic treatments they receive (Checcucci et al., 2020; Baker et al., 2022), exposure to co-selective compounds (Pal et al., 2017; Arya et al., 2021), and farm waste processing methods (Todman et al., 2024). Evidence highlights that while animal waste can introduce associated bacteria and ARGs directly into soil, the indigenous soil resistome can also be enriched, even when waste is derived from animals which have not received antibiotic treatment (Udikovic-Kolic et al., 2014). Consequently, exposure of livestock to antibiotics does not always correlate consistently with the presence of ARGs in the soil.

Many studies concerned with the dissemination of antimicrobial resistance in soils and crops following animal waste amendment place emphasis on swine and poultry; comparative studies of animal waste generally indicate that cattle waste harbors fewer and less diverse ARGs relative to poultry and swine waste (Qian et al., 2018; Duan et al., 2019; He et al., 2020; Xu et al., 2020; Li et al., 2025). Nonetheless, the abundance of ARGs in cattle waste can be comparable to that of hospital and municipal wastes (He et al., 2020), and may contain unique ARGs (Wichmann et al., 2014). Chávez-Fuentes et al. (2017) estimate that the global rate of organic matter production in cattle manure is at least five-times that of poultry or swine, making it the principal source of organic fertilizer introduced into the environment worldwide.

Several studies have demonstrated transient increases in the relative abundance of ARGs and/or resistance-associated mobile genetic elements (MGEs) in soil amended with cattle waste (Fahrenfeld et al., 2014; Hu H. W. et al., 2016; Nõlvak et al., 2016; Muurinen et al., 2017; Macedo et al., 2020). The duration of elevated ARG concentrations in soils post-amendment is variable. For instance, some studies suggest enriched ARGs approach or return to background levels within 2 months (Fahrenfeld et al., 2014; Muurinen et al., 2017); others demonstrate ARG elevations can persist for more than 5 months (Hu H. W. et al., 2016; Nõlvak et al., 2016). Tyrrell et al. (2023) found that the overall resistome and bacterial microbiome of soil amended with cattle, swine or poultry manure exhibits no significant change, yet transient enrichment of specific bacteria and ARGs was detected. Another study found the long-term application of cattle manure had minimal impact on the ARG profile of soil when compared to other livestock waste (Peng et al., 2017).

The majority of studies examining the influence of cattle waste on soil resistomes employ quantitative-PCR (qPCR) arrays (Hu H. W. et al., 2016; McKinney et al., 2018; Zhang et al., 2019; Tyrrell et al., 2023); sometimes in combination with 16S rRNA amplicon sequencing to assess bacterial community composition. This allows rapid quantification of hundreds of ARGs, but they are restricted to *a priori* selection. With sufficiently deep sequencing, shotgun metagenomics allows the full extent of known ARGs to be considered. In addition, assembling metagenome derived contigs can allow full-length ARG open reading frames to be recovered and associated with potential host organisms: the qPCR/amplicon approach relies on correlative inference of association. Metagenomes therefore provide an improved understanding of whether an ARG is circulating within animal waste, bacteria or typical soil community members.

Existing metagenomic studies involving cattle waste have largely been limited to single points in time (Noyes et al., 2016; Guron et al., 2019; Zhu et al., 2022) and rarely consider the temporal behavior of amendments, or of soil. Although Jauregi et al. (2021) employed metagenomics to investigate different dairy farm manure storage compartments (fresh and aged manure), these were only sampled once, giving a brief snapshot in time. Macedo et al. (2021) used targeted metagenomics to track the impact of dairy slurry on six different soils over a 21-day period, however, only a single application event was considered. Using qPCR, Hurst et al. (2019), investigated changes in the resistome of cattle slurry on several occasions over a year, however the implications for manure-amended field soil were not considered. In contrast, Liu et al. (2021) carried out a qPCR study characterizing the changing resistome of soils receiving either cattle, swine or poultry manure over a three-year period, however, the manure resistomes were not analyzed. Collectively, these studies demonstrate growing interest in the dynamics of manure- and soil-associated resistomes, yet most remain limited to single application events or short observation periods. In contrast, our study is unique in integrating multiple slurry application events with repeated soil and slurry sampling over an extended temporal scale, enabling assessment of both immediate and cumulative responses in the resistome and microbial community. This multi-event, multi-timepoint design provides a more realistic representation of how repeated slurry management practices shape soil microbial and resistance dynamics under field conditions.

In contrast, numerous microcosm studies have been conducted (Munir and Xagoraraki, 2011; Sandberg and LaPara, 2016; Gou et al., 2018; Chen et al., 2019); these allow for a highly controlled experimental regime, but are unlikely to capture seasonal factors operating at a farm-scale effectively, whether this relates to seasonal changes, waste management or meteorological shifts. Some studies have evaluated seasonal changes in soil resistomes (Xiang et al., 2021), however they do not incorporate concurrent temporal analysis of applied cattle waste. One exception is a metagenomic study during which field soil and cattle manure were collected over 2 years, however, manure and soil were only sampled in Autumn and Spring, providing relatively coarse insights into seasonal dynamics (Sukhum et al., 2021). Furthermore, Sukhum et al. (2021) indicated that the assessed farms applied manure once prior to soil collection during the study period. Temporal metagenomic studies are therefore lacking where multiple application events occur within a single year. Lastly, the absence of metagenomes for sites which have no history of systematic cattle manure exposure, yet remain geographically comparable, hinders the ability to identify the long-term impact of cattle waste exposure on the soil resistome and microbiome.

We have highlighted that at the farm-system level, temporal dynamics remain poorly integrated within existing research on the transfer and persistence of AMR in agricultural soil. Existing analyses of AMR in slurry-fertilized soil are largely unable to determine how seasonal changes in both slurry and field soil may influence the transfer and persistence of ARGs and their hosts in slurry-amended soil. Furthermore, few studies have followed field soil over multiple application events, as is typical on many farms. Consequently, it remains uncertain whether the resistome and microbiome of field soil respond uniformly to repeated application events throughout the year, as is common practice in high-intensity dairy farming. It is important that these ‘real-world’ factors are studied, if the relative risk associated with AMR in dairy farming is to be estimated accurately, understood, and mitigated.

We adopted a metagenomic approach, following changes in the resistome and bacterial microbiome of dairy cattle slurry and field soil over multiple slurry application events. We hypothesized that seasonal shifts would be observed in both slurry and field soil resistomes. We also expected to show a cumulative effect on the soil resistome following repeated slurry applications.

Concentrating sampling effort on a single dairy farm allowed us to produce a large library of metagenomes from which to reconstruct metagenome assembled genomes (MAGs) from slurry and field soil samples. We used these MAGs to track the persistence of slurry-borne bacteria introduced to soil. Finally, we used a nearby field site with no history of cattle slurry amendment to compare with a field site subject to long-term cattle slurry fertilization.

## 2 Materials and methods

### 2.1 Study site

The focus of the present study was a medium-sized, high performance dairy farm in England, UK which housed ~200 milking Holstein Friesian cattle at the time of sampling. Comprehensive descriptions of the farm layout, antibiotic usage and waste-handling practice are provided by Baker et al. (2022) and Todman et al. (2024). Cattle are housed indoors and resulting waste streams (feces, urine, antimicrobial footbath washings, rainwater run-off and antibiotic-contaminated milk) are directed into a 3,000 m^3^ slurry tank following liquid–solid separation. Thus, the slurry tank contains a cocktail of microorganisms and chemical compounds receiving regular input.

Slurry is periodically used to fertilize nearby grassland and arable fields. However, as the farm lies within a nitrate vulnerable zone (NVZ), slurry application is prohibited between October and January. An NVZ is defined as an area deemed at risk from agricultural nitrate pollution, and this designation is currently applied to over half of land in England (GOV.UK, 2025). Excess slurry produced during the ‘closed period’ is accommodated in an additional 8,000 m^3^ lagoon. An adjoining field with a long-term history of multiple, sub-annual slurry applications was selected to represent a slurry-treated grassland site (52^o^50’23.93”N, 1^o^14’38.82”W). The site is characterized by heavy clay loam topsoil and was maintained as a permanent grassland for the duration of the study. Grass was occasionally harvested prior to slurry application for silage production. Surface application of slurry at this location was carried out using an umbilical system connected to slurry tanker-mounted dribble-bars. Slurry was drawn from either the primary tank or overflow lagoon.

A nearby site with no documented history of systematic agricultural waste exposure was chosen to represent an untreated grassland site (52^o^50’1.64”N, 1^o^15’14.65”W). The untreated site is characterized by silty clay loam topsoil and has been maintained as a permanent grassland for more than 50-years. The two field sites share the same local climate; however, they are separated by both abiotic and biotic barriers including hedges, several rows of trees and a road, which we assume limits cross-exposure for the purposes of this study.

### 2.2 Sample collection

Soil samples were collected over an entire season of slurry application from the treated site (seven occasions between May 2017 and May 2018). For the May 2017 application, treated soil samples were collected 5 days before, within 24 h after, and 56 days following slurry application to quantify short-term temporal responses. These intervals were chosen to represent (i) baseline pre-application conditions, (ii) the immediate introduction of slurry-borne microorganisms and resistance genes, and (iii) the subsequent recovery period after short-term slurry effects typically diminish. Subsequent sampling dates focused on the longer-term effects of slurry amendment.

Sampling of the untreated site was carried out twice, once in January and May 2018 (corresponding samples were collected from the treated site on the same day). To maximize capture of local heterogeneity, three soil cores were taken from five locations arranged 20 m apart in a ‘W’ transect. All soil cores were taken using a 10 cm stainless steel auger sterilized with ethanol between each use. Cores were deposited immediately in sterile plastic resalable bags and transported to the laboratory within 2 hours. Upon arrival, each triplicate set of cores was combined and thoroughly mixed with a sterile spatula. Fifty g of soil from each composite sample was stored at −80 °C pending DNA extraction; the remainder was stored at 4 °C for physiochemical analyses (Supplementary File 1). Samples sizes were *n* = 35 (slurry fertilized site soil), *n* = 10 (untreated site soil).

Slurry tank samples were collected monthly between June and October 2017 (× 5 sampling occasions). Samples were collected using a clean stainless-steel bucket and aliquoted into two sterile glass bottles (*n* = 10) as described by Baker et al. (2022). A visual timeline of the sampling strategy is summarized in Supplementary Figure 1.

### 2.3 DNA extraction and sequencing

Environmental DNA was extracted from triplicate composite soil samples using DNeasy PowerSoil Kits (Qiagen) according to the manufacturer’s instructions. DNA quality and yield were determined by spectrophotometry (NanoDrop 1000, Thermo Scientific) and fluorometry (Qubit, Invitrogen), respectively and extraction replicates subsequently pooled.

High sensitivity DNA assays (Qubit, Invitrogen) for negative control DNA extractions performed on UltraPure DNase/RNase-Free Distilled Water (Invitrogen) were below the limit of detection. Sequencing and demultiplexing of soil sample extractions (*n* = 45) was performed by Edinburgh Genomics on an Illumina NovaSeq platform (150 bp paired end libraries). The current work considers a subset of 10 slurry tank metagenomic libraries reported by Baker et al. (2022). Slurry tank samples were extracted with QIAamp Powerfecal Kits (Qiagen) and sequenced by Edinburgh Genomics on an Illumina HiSeq platform. After sequencing, adapter sequences and low-quality reads were filtered with *TrimGalore* (v 0.4.4) using default settings. On average, 12 Gbytes of data were produced per sample. Metagenomic sequences were uploaded to the European Nucleotide Archive (ENA) under the following accession: PRJEB38990. Individual accessions and descriptions of samples analyzed in this study are detailed in Supplementary File 2. Note, sample S86 was excluded from all analyses due to suspected contamination. Therefore, final sample sizes included in analyses were *n* = 10 (slurry), *n* = 34 (slurry fertilized soil), and *n* = 10 (untreated soil).

### 2.4 Metagenome assembly

Short-read metagenomic libraries were subsequently assembled as individual samples using *Megahit* (v1.1.3) with ‘meta-large’ settings applied (*k*-mer range: 27–87, *k*-step = 10) and a minimum multiplicity for filtering of two. Three separate co-assemblies were also generated from slurry-treated soil, untreated soil and slurry metagenomes, respectively. For co-assemblies, a *k*-mer range of 29–89 (*k*-step = 10) and a minimum multiplicity for filtering of two was used.

#### 2.4.1 MAG generation and identification of slurry-associated MAGs

To investigate microbiomes of the sampled environments further, and identify potential slurry-borne bacteria in soil, co-assemblies were generated to recover metagenome-assembled genomes (MAGs) as follows. Co-assemblies were filtered to remove contigs shorter than 1 Kbp in length. Quality filtered reads from the slurry tank, treated and untreated site soil libraries were then mapped back to their corresponding co-assemblies using *BWA-mem* v0.7.17(r1188) (Li, 2013). To recover putative slurry-borne MAGs in treated soil metagenomes, slurry reads were also mapped against the treated soil site co-assembly. Slurry reads were also mapped against the untreated site soil co-assembly as a control. The output alignments were then converted into BAM format using *SamTools* v1.1.2 (Li et al., 2009). Binning was performed with *MetaBAT2* v2.12.1 (Kang et al., 2019). The quality of bins was assessed with *CheckM* v1.1.2 (Parks et al., 2015); only bins meeting an estimated quality score ≥ 50 as defined by Parks et al. (2017), were considered for further analysis. Further quality checking of MAGs of interest was performed using *MDMcleaner* (Vollmers et al., 2022). MAGs passing quality thresholds were classified taxonomically using *GTDB-Tk* v0.3.2 (Chaumeil et al., 2020) and compared using *dRep* v2.5.4 (Olm et al., 2017) (MAG taxonomic classifications are included in Supplementary File 3). Initial clustering of MAGs was performed with an applied threshold of 90% MASH ANI (average nucleotide identity), followed by secondary clustering with an ANImf threshold of 99%. The relative abundance of select MAGs of interest was calculated by mapping reads from each sample library to the MAG using *BWA-mem* and then dividing the number of aligned reads by the estimated bacterial genome number of each sample. To validate the presence of complete MAGs in individual samples further, we determined the distribution of mapped reads across each constituent contig belonging to the MAG using *CoverM* as described in Poff et al. (2021). The “contig” setting was used with the following cut-offs: 95% minimum identity and 75% of read bases minimum alignment length. Contig coverage was calculated using the “trimmed_mean” option. Contig coverage and length is displayed for each sample in Supplementary File 4.

#### 2.4.2 Antibiotic resistance gene annotation

For short-read metagenomic libraries, ARGs were annotated according to *ARG-OAP* (v2.0) using an ARG reference sequence identity cut-off of 80% and minimum query alignment length more than 25 amino acids, expect-value 1 × 10^−7^ (Feng et al., 2018; Murray et al., 2019; Qian et al., 2021). Normalization of ARGs by bacterial genome number was carried out by screening for 30 universal, single copy genes using *diamond* (v2.0.15), as implemented by *ARG-OAP*. ARG annotation in assembled contigs was performed with the resistance gene identifier platform (*RGI* v6.0.0) using the CARD database (v3.2.5). Only matches exceeding 90% identity and coverage of reference protein sequences were considered in analyses.

#### 2.4.3 Taxonomic classification of reads and contigs

Taxonomic classification of short-reads was performed using *Kaiju* (v1.7.1) with default settings (Menzel et al., 2016). A pre-built protein reference database was used (*nr_euk*, downloaded from the *Kaiju* webserver in June 2019) which contains a subset of non-redundant sequences from the NCBI BLAST *nr* database encompassing protein sequences of archaea, bacteria, viruses, fungi and protists.

#### 2.4.4 Statistical analysis of resistome and bacterial compositions

Overall differences between slurry, slurry-treated soil and untreated soil samples were considered. Differences between treated site soil samples collected 5 days prior to slurry application, within 24 h of application, and 56 days after slurry application in May 2017 were also assessed. We also identified differences between treated site soil and untreated site soil samples collected over the same period in January and May 2018.

Principal Coordinates Analysis (PCoA) was used to generate unconstrained multivariate ordinations of sample compositions. Hellinger distances of bacterial genome-normalized ARG abundance were used for resistome analyses, Bray–Curtis dissimilarity of genome-normalized taxon counts were used for bacterial community compositions. Where PCoA indicated clustering, permutational multivariate analysis of variance (PERMANOVA) was conducted to establish whether significant differences were observed between groups, following testing for heterogeneity of multivariate dispersion using the PERMDISP test. Linear relationships between sample type and ARGs or taxa (*r* > 0.4) were then identified using canonical analysis of principal coordinates (CAP). PCoA, PERMDISP, PERMANOVA and CAP were all implemented using the *PERMANOVA+* add on to *PRIMER* version 7.0.23. For all tests, probabilities were based upon 99,999 permutations (denoted *p*_perm_). In cases where the number of observations was insufficient to allow at least 100 permutations for *post hoc* pairwise tests, Monte Carlo probabilities (denoted *p*_MC_) were calculated based upon an asymptotic permutation distribution. PERMANOVA was thus used to test whether community composition differed significantly among sample types, while CAP was used to visualize and identify the variables most strongly associated with those differences.

Only ARGs or taxa with CAP group associations where *r* > 0.2 were further analyzed. To account for non-normal data distribution, Kruskal-Wallis tests were applied where three or more groups were compared, followed by pairwise Wilcoxon *posthoc* tests. The Benjamini-Hochberg procedure for false discovery rate adjustment was applied where multiple tests were applicable; *p*-values (denoted *p_adjust_*). Where only two groups were compared, a Mann–Whitney U test was performed. Where interaction effects were tested, a nonparametric factorial ANOVA was performed on aligned rank transformed data using the *ARTools* (v 0.11.1) R package. Significant interaction effects were further evaluated using the art.con contrasts function.

*p* values are reported in decimal format when ≥ 0.001, and in scientific notation when < 0.001 to improve readability. In some cases, both formats are provided (e.g., *p* < 0.001; = 4 × 10^−9^) to indicate both scientific threshold and exact value.

#### 2.4.5 Resistome and taxon diversity

We described diversity of taxa and ARGs using Hill numbers, with units of effective number of taxa or ARGs, respectively (Chao et al., 2014). Hill numbers were implemented to compare alpha diversity (representing diversity as effective numbers of taxa or ARGs, offering an approach that makes it easier to understand and communicate diversity levels compared to more abstract metrics such as Shannon or Simpson indices across resistome and microbiome datasets in a unified framework) (Alberdi and Gilbert, 2019). Different *r* thresholds were applied to resistome and microbiome CAP analyses to account for the differing levels of compositional complexity since ARG datasets were sparser and less even than taxonomic data.

These express diversity, *D,* weighted by abundance (*^q^D*) such that an unweighted estimate of *D* (i.e., taxon or ARG richness) is provided by ^0^*D* where abundances of individual taxa or ARGs are not considered; ^1^*D* where the taxa or ARGs are weighted proportional to their frequencies, representing the effective number of common or typical species (i.e., taxa or ARGs with typical abundances); and ^2^*D* which favors abundant species and does not consider rare species and so represents the effective number of dominant or highly abundant taxa or ARGs in each assemblage. Diversity estimates were generated for both taxa and ARGs using the abundance data produced by *Kaiju* (taxonomic profiles) and *ARG-OAP* (resistome profiles), analysed with iNEXT (v3.0.0). Extrapolation of *^q^D* values was extended to the larger of either the maximum sequencing depth across all samples or twice the number of sequences in the smallest sample.

For each sample type, differences between the mean extrapolated *^q^D* at different sampling time points were tested using a one-factor analysis of variance (ANOVA) employing the Welch adjustment of the denominator of the *F* ratio in cases where significant heterogeneity of within-group variance was determined using Levene’s test and basing probability upon 99,999 permutations where non-normal data distributions were identified by the Shapiro–Wilk test. To allow comparison between different *^q^D* estimates and different sources, we also calculated an estimate of treatment effect size, omega-squared (ω^2^) calculated according to $\omega^{2}=\frac{SS_{\mathrm{bg}-df_{\mathrm{bg}}}\cdot MS_{\mathrm{wg}}}{SS_{\text{total}}+MS_{\mathrm{wg}}}$ where *SS* represents the sum of squares, *MS* represents the mean square, *df* represents the denominator degrees of freedom, and the subscripts *bg* and *wg* represent between group and within group, respectively.

## 3 Results

### 3.1 The microbiome and resistome of slurry and soil are distinct

#### 3.1.1 Slurry tank resistome and bacterial composition

According to the average bacterial genome-normalized abundance of ARGs, the cattle slurry tank resistome was dominated by macrolide-lincosamide-streptogramin (MLS) resistance genes (0.24 mean copies per bacterial genome [cpbg], standard error of the mean ± 0.01). ARGs belonging to tetracycline (0.12 ± 0.003 cpbg), aminoglycoside (0.09 ± 0.01 cpbg) and beta-lactam (0.008 ± 0.05 cpbg) resistance gene groups were also prevalent in slurry. The normalized abundance of ARG subtypes reflected the category level trends, with *tetM* (tetracycline), *ugd* (peptide polymyxin), *lnu(H)* (MLS), *mel* (MLS), and *lnu(C)* (MLS), representing the five most abundant gene subtypes (Supplementary Figure 2A). Slurry samples were dominated by bacteria belonging to Firmicutes and Bacteroidetes (Supplementary Figure 3B).

#### 3.1.2 Soil resistome and bacterial compositions

**Figure 1:**
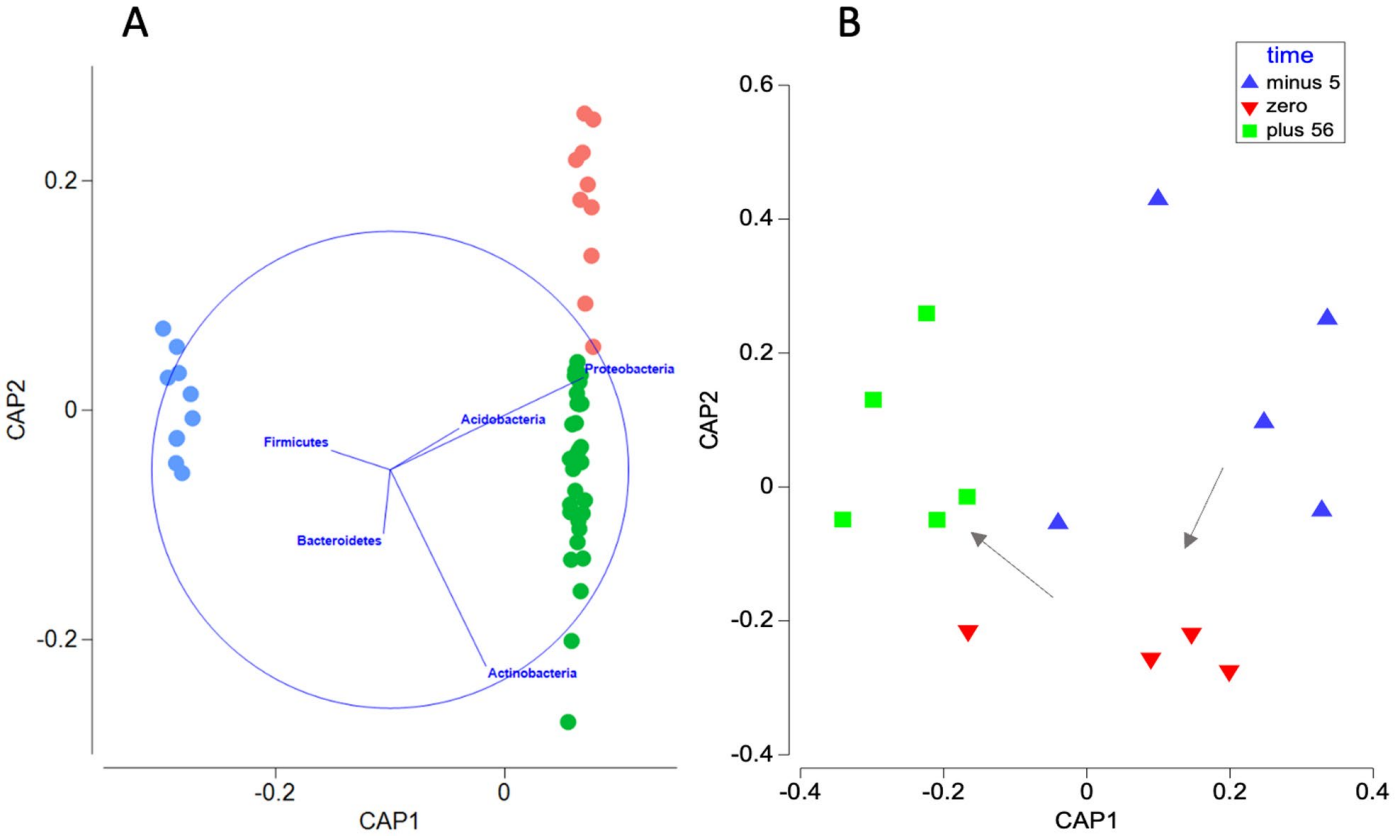
Discriminant canonical analysis of principal coordinates (CAP) of bacterial-genome normalized bacterial phylum abundance (Bray–Curtis dissimilarity) showing **(A)** separation of slurry tank (blue), slurry-treated site soil (green), and untreated site soil (red) bacterial phyla assemblages. First squared canonical correlation ( $\delta_{1}^{2}$ ) = 0.9988, $\delta_{2}^{2}$ = 0.6496. Sum of canonical eigenvalues = 1.65, *p_perm_* < 0.001; = 1 × 10^−5^. Mis-classification error of a leave-one-out cross validation = 1.85%. Vector overlays represent multiple partial correlations (*r* > 0.2) of the abundance of individual phyla with CAP axis scores. The length and direction of each vector indicates the strength and sign, respectively, of the relationship between that variable and the CAP axes. The circle is a unit circle (radius = 1.0), whose relative size and position of origin is arbitrary with respect to the underlying plot. **(B)** Discriminant CAP showing shifts in the composition of bacterial phyla in soil samples collected from the slurry-impacted site 5 days before (blue), < 24 h after (red) and 56 days after (green) the first slurry application of May 2017. $\delta_{1}^{2}$ = 0.717, $\delta_{2}^{2}$ = 0.585. Sum of canonical eigenvalues = 1.30, *p*_perm_ < 0.05; = 3.1 × 10^−2^. Mis-classification error of a leave-one-out cross validation = 21.4%. Arrows indicate the temporal sequence of sampling.

The soil resistome was distinct from that of cattle slurry, irrespective of whether samples were derived from the site with a history of repeated slurry fertilization or not. Untreated and slurry-treated soil resistomes were dominated by multidrug resistance genes (0.06 ± 0.001 cpbg), bacitracin (0.04 ± 0.0007 cpbg), rifamycin (0.03 ± 0.0008 cpbg) and polymyxin peptide (0.01 ± 0.0002 cpbg) ARGs. A single bacitracin peptide resistance gene group *bacA* dominated field soils, and several prevalent ARG subtypes were shared across the sites; *mexF* (multidrug efflux), *rphB* and *rbpA* (rifamycin) (Supplementary Figures 2B,C). Soil from the treated and untreated sites were both dominated by members of the Proteobacteria and Actinobacteria (Supplementary Figure 3B). However, CAP overlay vectors *r* > 0.2 (Figure 1A), combined with Mann–Whitney tests of treated and untreated soil samples indicate that Actinobacteria were more prevalent in treated site soil (*p*_adjust_ < 0.001; = 1 × 10^−4^), while Proteobacteria (*p*_adjust_ < 0.001; = 1 × 10^−5^) and Acidobacteria were enriched in untreated soils (*p*_adjust_ < 0.05; = 4.52 × 10^−2^).

### 3.2 Long-term slurry application alters field soil microbiomes, but typical soil bacterial phyla and ARGs remain dominant

**Figure 2:**
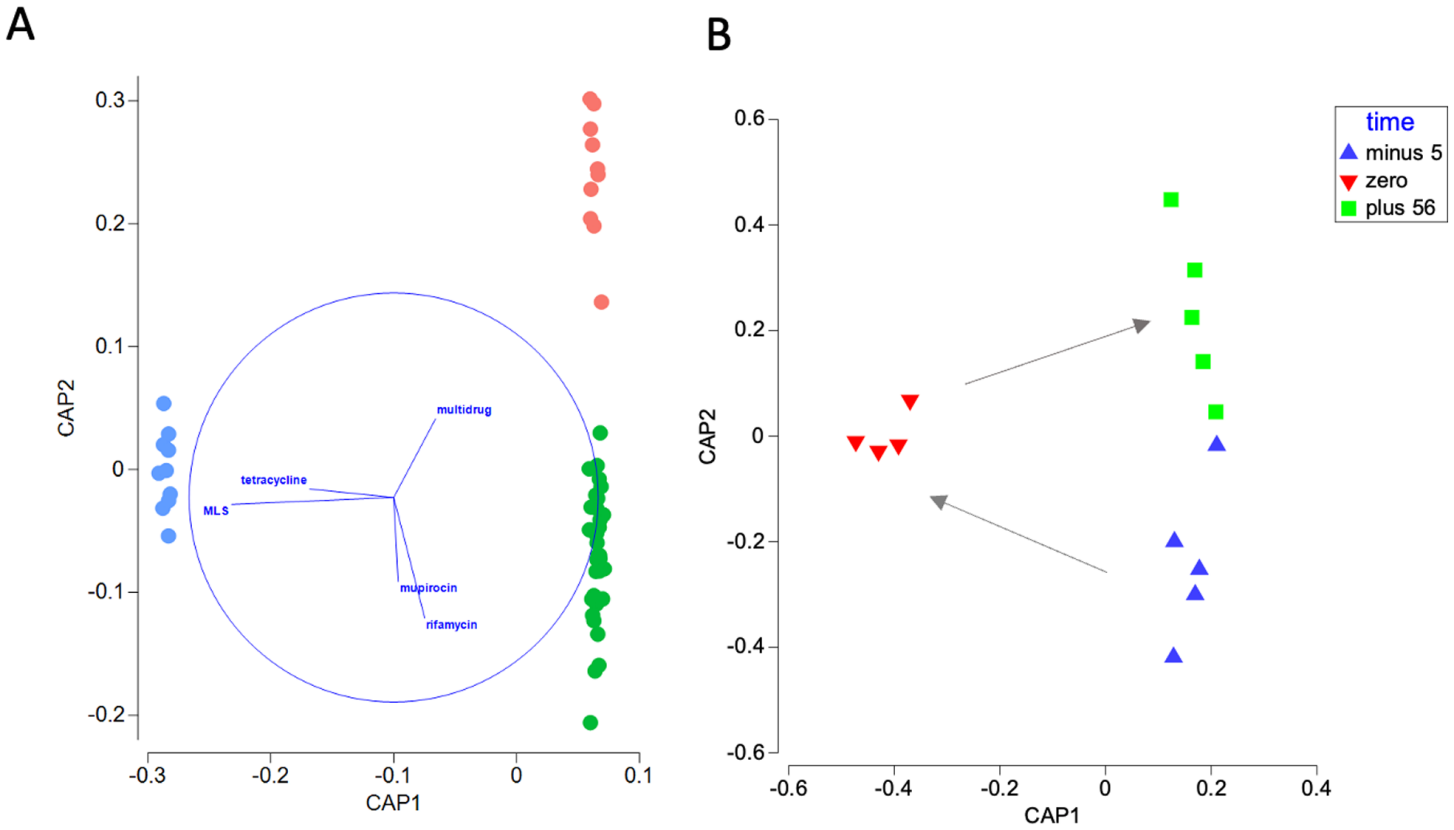
**(A)** Discriminant CAP of sample resistomes based upon bacterial genome-normalized ARG abundance (Hellinger distance). The resulting ordination distinguishes between ARG assemblages in the two field sites (slurry-treated soil = green, untreated soil = red) and slurry (blue). First squared canonical correlation ( $\delta_{1}^{2}$ ) = 0.9995, $\delta_{2}^{2}$ = 0.8589. Sum of canonical eigenvalues = 1.86, *p_perm_* < 0.001; = 1 × 10^−5^. Mis-classification error of a leave-one-out cross validation = 0%. Vector overlays represent multiple partial correlations (*r* > 0.4) of the abundance of individual antibiotic resistance gene categories with CAP axis scores. The length and direction of each vector indicate the strength and sign, respectively, of the relationship between that variable and the CAP axes. The circle is a unit circle (radius = 1.0), whose relative size and position of origin is arbitrary with respect to the underlying plot. **(B)** CAP showing shifts in the composition of ARG categories in soil samples collected from the slurry-impacted site 5 days before (blue), < 24 h after (red) and 56 days after (green) the first slurry application of May 2017. First squared canonical correlation ( $\delta_{1}^{2}$ ) = 0.9851, $\delta_{2}^{2}$ = 0.7474. Sum of canonical eigenvalues = 1.73, *p_perm_* < 0.01; = 2 × 10^−1^. Misclassification error of a leave-one-out cross validation = 21.4%. Arrows indicate the temporal sequence of sampling.

Statistical analyses based on CAP vectors *r* > 0.4 indicated that tetracycline (Mann–Whitney *p*_adjust_ < 0.05; = 2.39 × 10^−2^), MLS (Mann–Whitney, *p*_adjust_ < 0.01; = 6.8 × 10^−3^), mupirocin (Mann–Whitney, *p*_adjust_ < 0.001; = 3 × 10^−7^) and rifamycin (Mann–Whitney, *p*_adjust_ < 0.001; = 5 × 10^−5^) ARGs distinguished treated and untreated soils. After correction for multiple testing, multidrug ARGs (Mann-Witney, *p*_adjust_ > 0.05; = 8.5 × 10^−2^) were not significantly different between the two soils (Figure 2A).

The average normalized abundance of total detected ARGs was significantly different between sites (Kruskall Wallis *χ*^2^ = 27.4, *p* < 0.001; = 1 × 10^−6^). Specifically, total ARG abundance was greater in cattle slurry (0.60 ± 0.01 cpbg) relative to both treated (Pairwise Wilcoxon, *p*_adjust_ < 0.001; 2 × 10^−9^) and untreated (Pairwise Wilcoxon, *p*_adjust_ < 0.001; = 2 × 10^−5^) soil. There was a significant difference between the soil of the treated (0.25 ± 0.001 cpbg) and untreated site (0.23 ± 0.007 cpbg) (Pairwise Wilcoxon, *p*_adjust_ < 0.05; = 2.25 × 10^−2^).

PERMANOVA applied to Hellinger distances of the ARG category data suggested sample group had a significant influence on ARG compositions (pseudo-*F* = 582.3, *p*_perm_ < 0.001; = 1 × 10^−5^), contributing to approximately 19% of overall resistome variation. Furthermore, pairwise comparisons showed that the slurry resistome was significantly different from the soil samples, irrespective of site (treated site, *t* = 37.1, *p*_perm (adjust)_ < 0.001; = 1 × 10^−5^; untreated site, *t* = 24.0, *p*_perm (adjust)_ < 0.001; = 1 × 10^−5^), and that despite the high-level similarities hitherto described, soil samples from the treated field were also significantly different from those collected from the site with no history of slurry application (*t* = 4.0, *p*_perm (adjust)_ < 0.001; = 1 × 10^−5^). Nonetheless, CAP shows that both field site soil resistomes cluster together on axis 1, with separation primarily on axis 2 (Figure 1A). This was mirrored by bacterial taxa at the phylum level (pseudo-*F* = 3183.5, *p_perm_* < 0.001; = 1 × 10^−5^) (for pairwise comparisons see Supplementary Table 1).

### 3.3 Slurry application induces immediate short-term changes in the resistome

To understand the immediate impact of slurry application on the resistome of soil with a history of regular exposure, PERMANOVA was applied to a subset of treated site soil samples collected before and after the first application of slurry in May 2017 when 60 m^3^ ha^−1^ of slurry was applied. These samples correspond to a soil time series collected 5 days before, within 24 h of, and 56 days after, slurry application. Time had a significant influence on the resistome of these treated soil samples (PERMANOVA, pseudo*-F* = 2.4, *p*_perm_ < 0.001; = 7 × 10^−4^). At the ARG category level, pairwise comparisons indicated that the resistome was altered significantly on the day of slurry application relative to 5 days before (*t* = 1.7, *p*_perm (adjust)_ < 0.05; = 1.2 × 10^−2^). Additionally, the resistome composition within 24 h of slurry application was significantly altered compared to samples obtained 56 days later (*t* = 1.8, *p*_perm (adjust)_ < 0.05; = 1.2 × 10^−2^). However, the resistome of samples collected 56 days after slurry application was not significantly different from the pre-application samples (*t* = 1.2, *p*_perm (adjust)_ > 0.05; = 2.05 × 10^−1^), suggesting a transitory change in state. Differences were also identified in bacterial phyla compositions over the same period (pseudo-*F* = 4.7117, *p_perm_* < 0.01; = 2.5 × 10^−3^). However, according to pairwise comparisons, bacterial phyla compositions remained distinct from pre-application soil assemblages 56 days after slurry application (Figure 1B; Supplementary Table 1).

To investigate the enrichment of slurry-associated ARGs in soil, we conducted targeted analysis of MLS and tetracycline resistance genes, as these were shown to dominate the slurry resistome (Figure 2A; Supplementary Figure 2A). Furthermore, these ARG categories were among CAP vectors (*r* > 0.4) associated with soil samples < 24 h and 56 days after the first application of slurry in 2017, respectively (Supplementary Figure 4). Kruskall-Wallis tests showed that the normalized abundance of tetracycline ARGs (*χ*^2^ = 4.17, *p_adjust_* > 0.05; = 1.24 × 10^−1^) did not change significantly, whereas the abundance of MLS ARGs did change over the same period (*χ*^2^ = 7.43, *p_adjust_* < 0.05; = 4.88 × 10^−2^). Therefore, only MLS ARGs were considered in *post hoc* tests.

**Figure 3:**
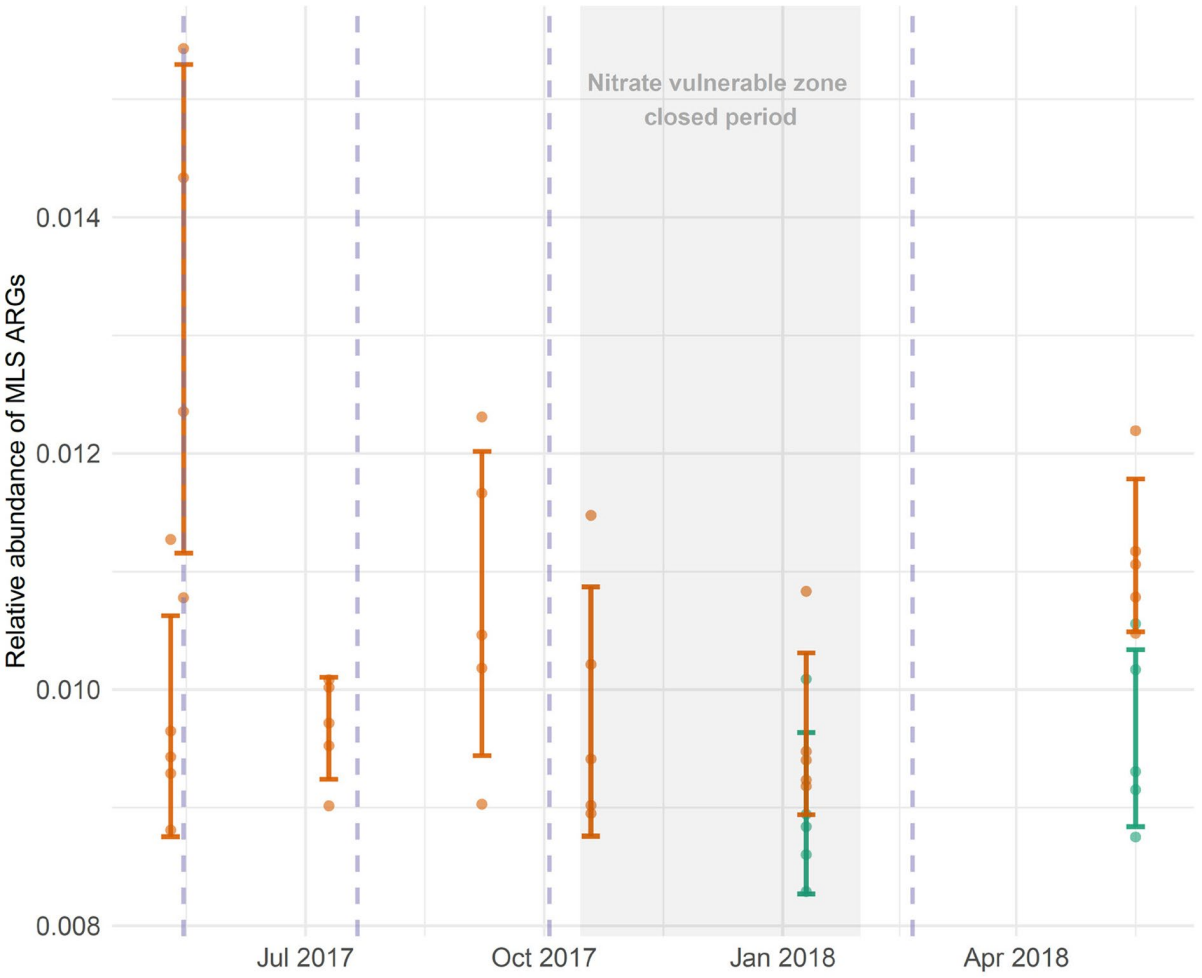
The bacterial genome-normalized abundance of macrolide, lincosamide, and streptogramin (MLS) antibiotic resistance genes (ARGs) (orange bars) were transiently increased after the first application of slurry in May 2017. Vertical dashed lines (pale purple), indicate when slurry was applied to the field; there were therefore four applications on the treated site over the course of the study. No slurry was applied during the ‘closed period’ because the farm is within a nitrate vulnerable zone (NVZ). Data for the control site (where no slurry was applied) are also shown for comparison (green bars). These control data are for January and May 2018.

Pairwise Wilcoxon tests show that the relative abundance of MLS ARGs was significantly different < 24 h after the first slurry application of 2017 (*p*_adjust_ < 0.05; = 4.76 × 10^−2^, Figure 3). However, pairwise comparisons of soil samples show that MLS ARGs declined to pre-application levels (pre-application soil comparison to 56 days after slurry application: *p_adjust_* > 0.05; = 5.48 × 10^−1^; Figure 3).

### 3.4 Seasonal shifts enrich MLS genes in field soil, irrespective of long-term slurry application

Analysis of MLS ARGs in January and May 2018 showed that the relative abundance of MLS ARGs was significantly different in the untreated soil compared to the slurry-treated soil in both January and May (ART- ANOVA, *F* = 10.31, *p* < 0.01; = 5.5 × 10^−3^). The relative abundance of MLS ARGs was also significantly different in January compared to May, regardless of treatment (ART- ANOVA, *F* = 10.25, *p* < 0.01; = 5.6 × 10^−3^), however, no significant interaction effect was observed between site and month (ART- ANOVA, *F* = 3.03, *p* > 0.05; = 1.01 × 10^−1^), as MLS ARGs increased in relative abundance from January to May in both locations. However, these genes were more abundant in the treated site overall (Figure 3).

#### 3.4.1 Slurry resistome richness is not tied to bacterial richness

Extrapolated Hill number-based estimates of taxon and ARG diversity were generated for slurry, and for both slurry-treated and untreated soils. Significant variation in ^0^*D* (richness) of both taxa (ANOVA, *F*_4,95_ = 20.8, *p*_perm_ < 0.001; = 1 × 10^−5^) and ARGs (Welch ANOVA, *F*_4,37.09_ = 25.0, *p* < 0.001; = 4.1 × 10^−10^) were observed in slurry samples, depending on the time of sampling. For taxa, ^0^*D* increased from June to a maximum in August. In contrast, highest ARG ^0^*D* were observed in June and diversity decreased to a minimum in August. Thus, there appears to be an inverse relationship between taxon and ARG richness in the slurry tank.

#### 3.4.2 Soil with no history of slurry application shows more dynamic changes in both resistome and bacterial taxon diversity than soil with long-term slurry exposure

**Figure 4:**
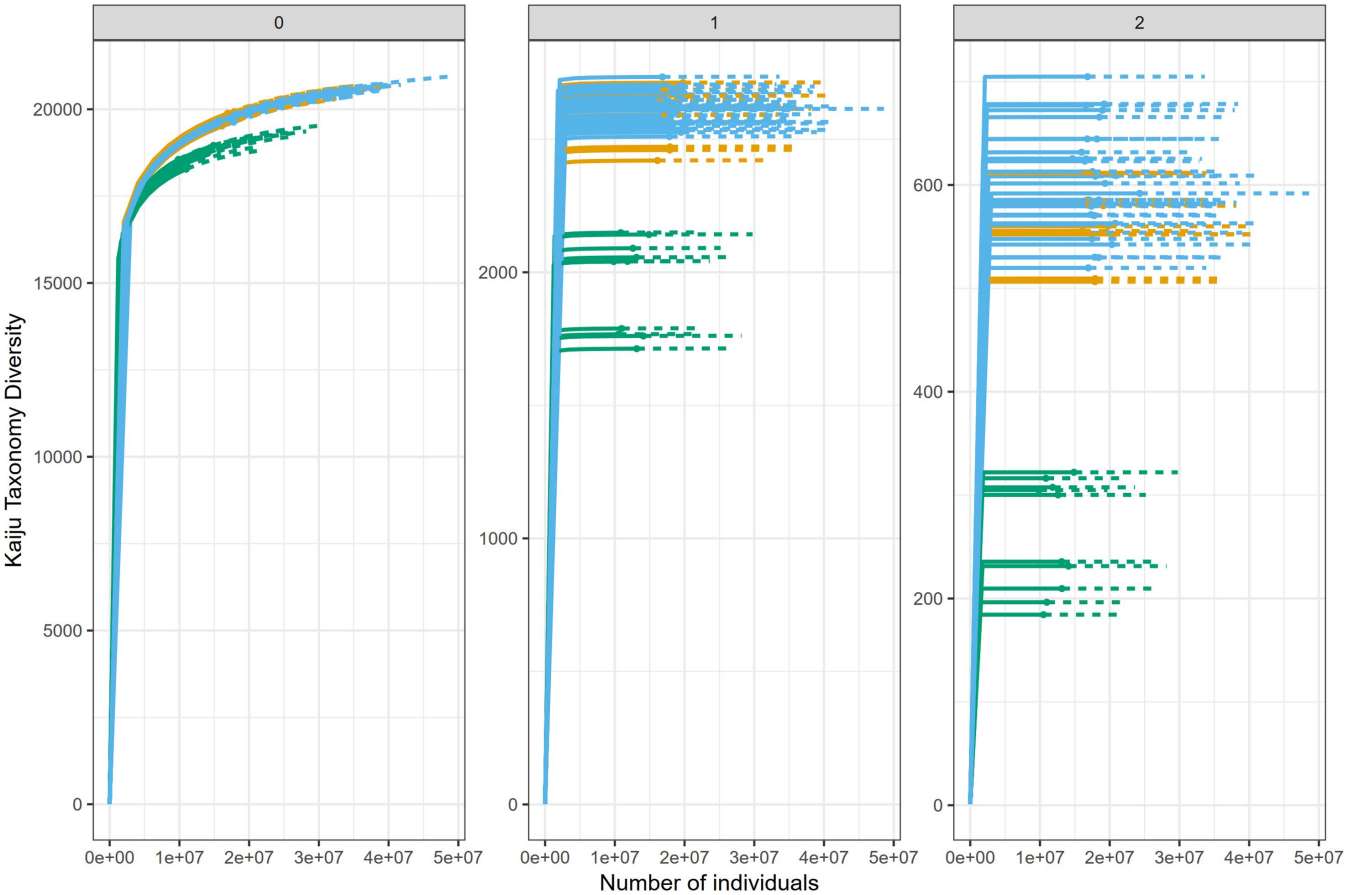
iNEXT diversity estimates for bacterial taxa, showing the greater diversity of soil relative to slurry. Color denotes sample source as follows: slurry (green), treated site soil (blue), and untreated site soil (orange). Solid line denotes observed values, dashed line denotes extrapolations.

**Figure 5:**
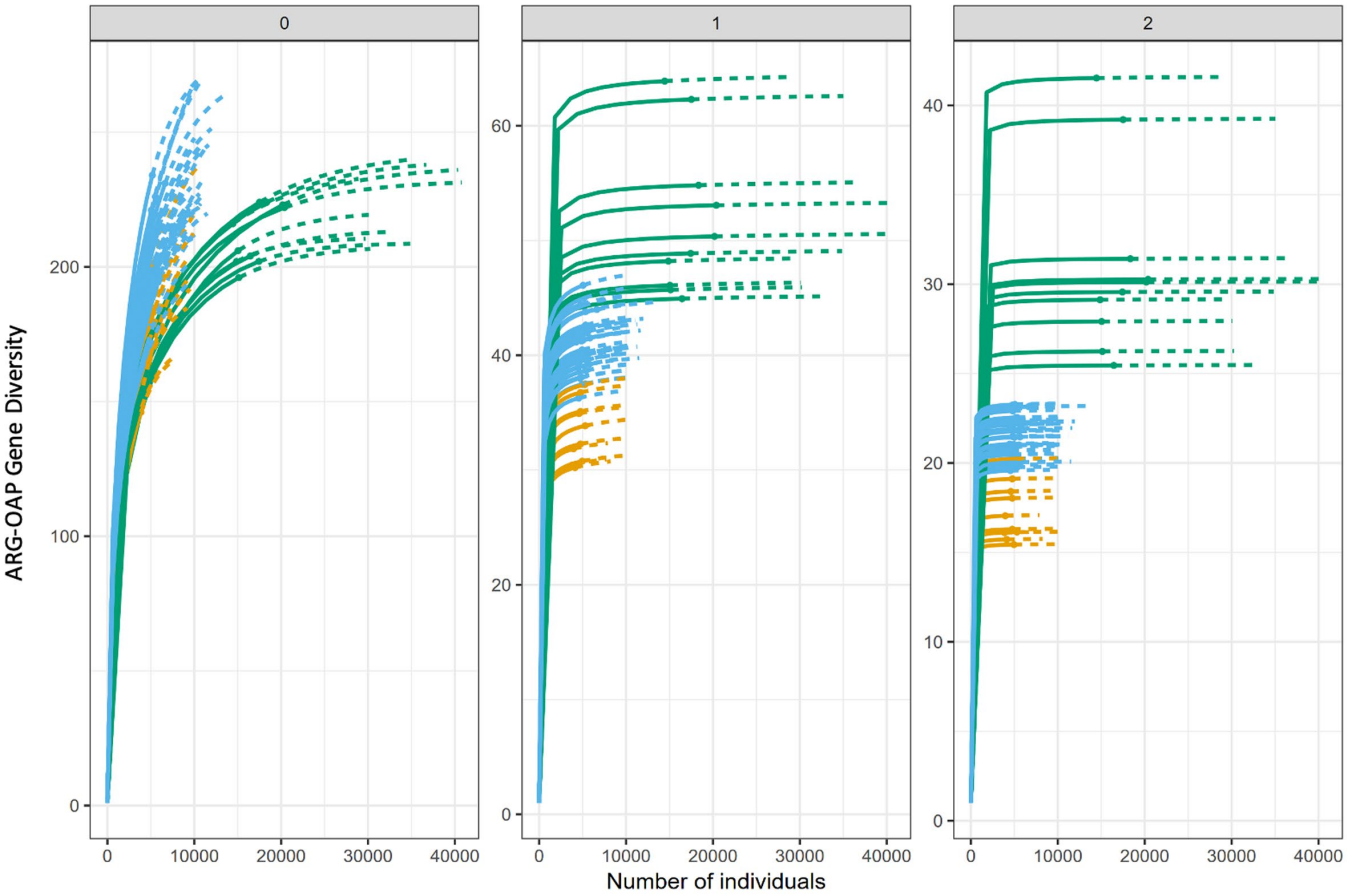
iNEXT diversity estimates for antibiotic resistance genes (ARGs) detected by ARG-OAP, showing the greater diversity of soil relative to slurry in q0 (richness), while iNEXT shows that the slurry has a greater diversity when reviewing dominant ARGs (q2). Color denotes sample source as follows: slurry (green), treated site soil (blue), and untreated site soil (orange). Solid line denotes observed values, dashed line denotes extrapolations.

In soils, there was little consistency in the behavior of taxonomic and ARG *α*-diversity (Supplementary Table 2). In untreated soil, bacterial taxonomic richness (*^0^D*) did not differ significantly between January and May, whereas ARG richness increased markedly over the same period (*p* < 0.01), indicating that ARG richness was more dynamic than bacterial richness. A similar pattern occurred in slurry-treated soil, where sampling month had little effect on taxon *^0^D* but significantly influenced ARG *^0^D*. Across the full sampling period, taxon *^0^D* was comparable between the two soils (Figure 4), while ARG richness was higher in slurry-treated soil (*p* < 0.001), though the effect size was small (Figure 5).

For the effective number of common taxa or genes (*^1^D*), differences between taxa and ARGs were more pronounced. In untreated soil, taxon *^1^D* showed a small seasonal increase, while ARG *^1^D* declined sharply from January to May, again demonstrating evidence of greater ARG dynamism. In contrast, slurry-treated soil displayed strong month-to-month variability in both taxon and ARG *^1^D*, but without consistent trends across the year.

Patterns associated with the most dominant taxa or ARGs (*^2^D*) followed a similar trend: diversity declined significantly between January and May in untreated soil, with larger effect sizes for ARGs than taxa. In slurry-treated soil, both taxon and ARG *^2^D* varied by sampling month, but seasonal effects were weaker than in untreated soil.

Collectively, these results demonstrate that ARG diversity was consistently more temporally dynamic than taxonomic diversity, particularly in untreated soil. In untreated soil, changes in microbial diversity were mainly driven by shifts among dominant taxa, while increases in ARG richness reflected the appearance of low-abundance ARGs. In contrast, diversity changes in slurry-treated soil were more associated with variation among common bacterial taxa and the most abundant ARGs, suggesting that long-term slurry inputs dampened seasonal fluctuations in the resistome.

#### 3.4.3 Putative recovery and temporal dynamics of a slurry-borne *Proteiniphilum* MAG

**Figure 6:**
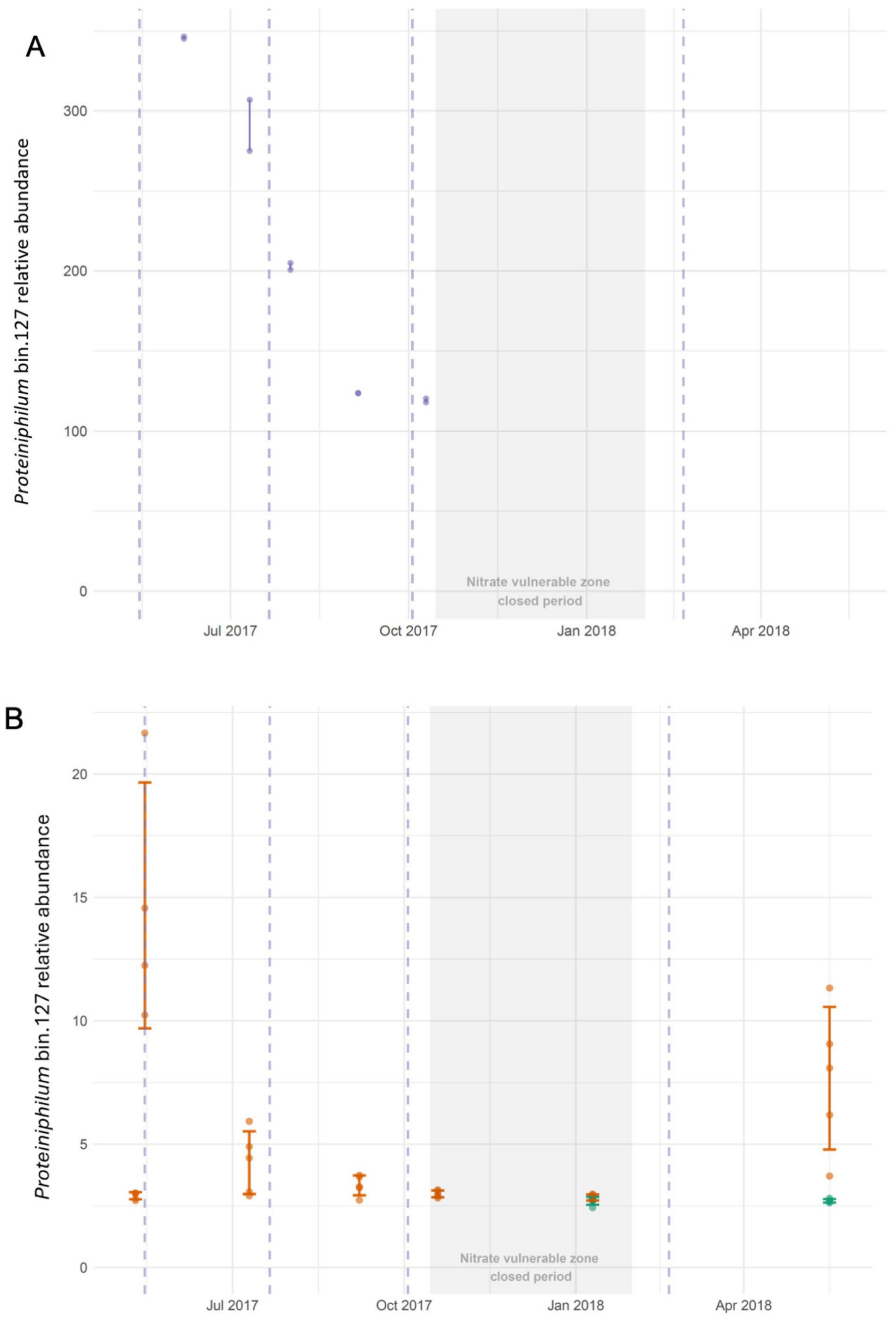
**(A)** *Proteiniphilum* MAG (bin 127) is a slurry borne organism which declined in abundance over the course of the year in the slurry tank (normalized by bacterial genome). Vertical lines are range bars. In the context of the slurry tank, the NVZ (closed period) refers to slurry storage since the tank will not be emptied during this period. **(B)** *Proteiniphilum* MAG (bin 127) transiently increased in soil immediately after slurry application in May 2017, but declined within 8 weeks and did not appear to be enriched at any point during the remainder of the year despite repeated slurry application. However, 8 weeks after the first slurry application of 2018 another spike in abundance was detected (while no such spike was identified in a nearby field not receiving any slurry amendment – green line). This suggests a dynamic response from the autochthonous soil community or other seasonal environmental factors which affect *Proteiniphilum* spp. survivability. Vertical dashed lines (pale purple), indicate when slurry was applied. No slurry was applied during the ‘closed period’ because the farm is within a nitrate vulnerable zone (NVZ). Vertical lines show confidence intervals.

A total of 164 MAGs (≥ 50 quality score) were recovered across cattle slurry (*n* = 136) and soil co-assemblies from treated (*n* = 15) and untreated (*n* = 13) sites. These were predominantly bacterial (*n* = 156) and a smaller number of archaeal MAGs (*n* = 9), including six slurry-associated methanogens (Supplementary File 3). Dereplication (≥ 90% and ≥ 99.5% ANI) identified a single cluster of three *Proteiniphilum* sp. MAGs: one from the slurry co-assembly (bin 127), one from the slurry-treated soil co-assembly (bin 293), and one obtained by binning slurry reads mapped to treated-soil assemblies (bin 4). No equivalent MAGs were recovered from untreated-soil assemblies, even after mapping slurry reads. *MDMcleaner* estimates suggested 60–75% completeness, with bin 127 (75% complete, slurry-derived) selected for downstream abundance profiling. No ARGs were detected in any *Proteiniphilum* MAGs. *Proteiniphilum* bin 127 abundance differed significantly across sites (Kruskal-Wallis χ^2^ = 35.146, *p* < 0.001), being highly enriched in slurry (216.43 ± 30.10 cpbg) relative to treated soil (5.26 ± 0.73 cpbg) and untreated soil (2.70 ± 0.04 cpbg) (pairwise Wilcoxon *p*_adjust_ < 0.001), confirming its slurry origin (Figures 6A,B). Following slurry application in May 2017, abundance increased significantly in treated soil within 24 h of slurry application (*p*_adjust_ < 0.05), and showed significant effects of site, month, and their interaction (ANT-ANOVA, all *p*_adjust_ < 0.01), with enrichment observed only at the treated site by May 2018 (~12 weeks post-application) (*p*_adjust_ < 0.01). While initial slurry additions in both years produced measurable short-term enrichment of *Proteiniphilum* sp. in treated soil, repeated applications during 2017 did not generate cumulative increases (pairwise Wilcoxon *p* > 0.05; Figure 6B). A declining trend in slurry supply was observed but was not statistically significant (Kruskal-Wallis χ^2^ = 8.73, *p* > 0.05), likely reflecting limited sampling power (Figure 6A). For a full table of contrasts, please see Supplementary Table 3.

## 4 Discussion

Using a medium-sized dairy farm operating in an area designated by the U. K. Department for Environment, Food and Rural Affairs as being at risk of water pollution from agricultural nitrate (termed a nitrate vulnerable zone), we investigated the impact of repeated dairy cattle slurry application events on the bacterial microbiome and resistome of grassland field soil.

### 4.1 The core bacterial community and resistome of grassland soil is largely resilient to bacteria and ARGs introduced by cattle slurry application

We detected significant shifts in the resistome and bacterial phyla immediately after the first application of slurry in the 2017 season. Although ARG compositions returned to their original states within 8 weeks of this application event, the composition of bacterial phyla remained altered for longer. Nonetheless, slurry-associated taxa and ARGs were not significantly enriched by the end of the 2017 application period, suggesting that the two subsequent slurry applications of that season did not result in any cumulative elevation of slurry-associated bacteria or ARGs. This is consistent with a previous metagenomic study which found limited evidence of persistent ARG transfer from dairy cattle manure to field soils (Sukhum et al., 2021).

The dominant ARG subtypes detected in the slurry-treated field site also dominated the untreated soil and showed comparable relative abundances, indicating repeated slurry applications had not altered the core resistome structure (Supplementary Figures 2B,C).

### 4.2 Long-term slurry application can enrich indigenous soil ARGs

Nonetheless, CAP highlights that the overall beta-diversity of ARG categories and bacterial phyla of soil receiving long-term slurry amendment were distinct from the control field site (Figures 1A, 2A). In terms of ARGs, CAP vector overlay (*r* > 0.4) and statistical tests indicate this was primarily due to the elevation of rifamycin and mupirocin ARGs in the treated site (Figure 2A).

Rifamycin and mupirocin resistance gene categories were not prevalent in slurry samples. No increase in these ARGs in soil was shown to coincide directly with slurry application events, suggesting their enrichment does not arise from direct introduction of genetic material via slurry. Rifamycin resistance genes such as *rphB* and *rbpA* were among the most abundant ARGs in soil samples, irrespective of amendment history and are most likely encoded by indigenous soil bacteria. Rifamycin B was first described in a soil actinomycete (Verma et al., 2011), and *rbpA* is known to occur frequently in Actinobacteria (Dey et al., 2012). The *rbpA* gene was also the only ARG to be recovered from soil metagenome assembly contigs; given that the soil was not sequenced to saturation, their recovery demonstrates their inherent abundance in soil. Nucleotide megaBLAST of the longest representative contig (S121_k87_335727, 1.2 kbp) indicated they were borne chromosomally within environmental *Mycobacterium* spp. (closest match *Mycobacterium* sp. ITM-2016-00318 accession CP134400.1, 97% query cover, nucleotide identity similarity 88.1%). Considering this, the greater abundance of rifamycin ARGs in the slurry-treated field site is most likely the result of long-term application which caused shifts in the resident bacterial community, leading to the enrichment of intrinsically resistant taxa (e.g., Actinobacteria; see Figure 1A). In contrast, the most abundant mupirocin ARG in both field sites was the *ileS* gene intrinsic to *Bifidobacteria*, a genus which typically inhabits gastro-intestinal tracts of animals. This ARG was an order of magnitude less abundant in cattle slurry relative to grassland soils, which was unexpected as it was shown to associate with cattle waste rather than soil in a previous study (Kang et al., 2022). The apparently high recovery of this ARG in field soil with no history of manure amendment may instead be explained by the presence of wild mammals.

### 4.3 Slurry application can promote short-term enrichment of MLS ARGs associated with slurry

We found that MLS ARGs dominated slurry tank resistomes together with tetracycline resistance genes. MLS genes are identified routinely among the core resistome of cattle waste and associated field run-off (Hu Y. et al., 2016; Noyes et al., 2016; Gou et al., 2018; Kang et al., 2022). There are no farm records of MLS antibiotics being administered to the milking herd (Baker et al., 2022), but tulathromycin (a macrolide antibiotic) was occasionally administered to calves, whose waste does not enter the tank. The occurrence of MLS ARGs is therefore unlikely to be connected to antibiotic use. Tetracycline ARGs are also known to be abundant in cattle waste (Kang et al., 2022; Shen et al., 2024) and were among the most commonly used class of antibiotics on the farm alongside beta-lactams. However, MLS and tetracycline ARGs may be enriched in cattle during early life due to dietary influences on microbial recruitment in the gut (Liu et al., 2019). Of these two categories, only MLS ARGs were significantly increased in soil immediately after the first slurry application of 2017 and they rapidly declined toward pre-application levels.

The transient enrichment of select MLS resistance genes in cattle-waste amended soils has been documented in previous field (Fahrenfeld et al., 2014; Muurinen et al., 2017) and microcosm (Chen et al., 2019) studies. Competitive action of indigenous soil microbiota is proposed to constrain the distribution and persistence of ARGs introduced to soil. Several laboratory-scale studies appear to support this (Peng et al., 2016; Chen et al., 2017; Klümper et al., 2019; Pérez-Valera et al., 2019). In contrast, at least one qPCR-based study has shown that MLS ARGs not only increase following decades of annual cattle waste application, but also remain elevated for over a decade after manure application has ceased (Zhang et al., 2023). Furthermore, in the study by Zhang et al. (2023), manure was applied only once per year. In another qPCR study by Macedo et al. (2020), soil was collected from experimental field sites of contrasting texture before and after three successive applications of cattle manure. Macedo et al. (2020) used the data to infer that select ARGs would decline to pre-application levels within 29–42 days. This implies that while a year-long interval could lead to an enriched MLS resistome in one study, select MLS ARGs (*ermB*) have also been shown to decline over much shorter time-periods.

Environmental factors, both abiotic and biotic, are therefore likely to contribute to the variation observed in the literature. Solid and liquid cattle waste have distinct chemical and biological properties which can affect their influence on soil microbial communities (Neufeld et al., 2017); environmental conditions, such as exposure to solar radiation, and rainfall may dramatically reduce or promote the survival of introduced bacteria (Hodgson et al., 2016; Jang et al., 2017). The rate and frequency of application on farms can be variable and the waste material itself may have variable bacterial loads, even if their community structures remain relatively stable. Hurst et al. (2019) found general seasonal trends in cattle manure-associated ARGs and demonstrated site-specific differences. In the present work, the relative abundance of MLS ARGs declined in the slurry tank from May to October 2017 (Supplementary Figure 5), which may explain why soil collected only 2 weeks after slurry application in October showed no apparent elevation in slurry ARGs. However, we note that previous analysis of the slurry indicated that the core resistome (including MLS ARGs) did not change significantly over the course of sampling (Baker et al., 2022). Nonetheless, in samples collected 84 days after the first slurry application of 2018, a significant elevation in MLS ARGs was observed compared to pre-application abundance in January (Figure 3). If this were due to slurry application, it would suggest that changes in either the slurry (unfortunately not sampled at this time) or the prevailing conditions in the field environment were able to promote the enrichment of these ARGs over a much longer period than shown after preceding applications, indicating considerable temporal variability in resistome behavior can exist within the same farming system.

Accordingly, it could be argued that this finding merely represents seasonal shifts in macrolide ARGs due to factors unrelated to slurry application. In a watershed-scale study the abundance of ARGs in soil significantly increased in spring relative to all other seasons (Xiang et al., 2021). This is also the time at which organic fertilizer is generally applied to enhance crop growth. We note that although samples collected from the treated site during May show an increase in MLS ARGs, a significant increase was also observed in the site with no history of cattle slurry amendment. Nevertheless, specific MLS subtypes associated with slurry such as *lnu(H)* were detected in treated field soil during May 2018, but were undetectable in all untreated site samples collected. Although our data indicate that the survival of slurry ARGs may be extended during different times of the year, especially spring, the small untreated soil sample size and lack of concurrent slurry samples require that we are cautious in our interpretation. Further multi-year, multi-farm studies are needed to confirm this finding.

A further observation from this study is that ARG alpha-diversity exhibited greater temporal variability than bacterial taxonomic alpha-diversity, particularly in untreated soil. This decoupling between resistome and microbiome dynamics suggests that ARG composition can fluctuate independently of major shifts in community structure. One possible explanation is that many ARGs, particularly those occurring at low abundance, are subject to rapid ‘input-decay’ cycles driven by transient introduction via environmental sources and subsequent loss through degradation or dilution. In contrast, taxonomic diversity tends to be stabilized by dominant and resilient soil taxa that maintain community structure across seasons. The higher responsiveness of ARG diversity to short-term environmental changes implies that the resistome may be a more sensitive indicator of AMR risk than overall microbial diversity. For on-farm management, this finding highlights the importance of temporal monitoring: infrequent or single-time sampling may underestimate the true dynamism of ARG pools, while management practices that reduce seasonal inputs or promote conditions favoring microbial stability could help moderate resistome variability.

### 4.4 Independent recovery of near-identical *Proteiniphilum* sp. MAG in slurry and slurry-treated soil highlights the utility of MAGs in microbial transfer analyses

To our knowledge, we provide one of the few examples where MAGs have been used to demonstrate the presence of a slurry-borne organism in slurry-amended field soil. A key finding of this study was the assembly of a *Proteiniphilum* sp. MAG from the slurry-receiving soil. This shared high sequence homology (> 99.5% ANI) with another MAG retrieved independently from slurry metagenomes. We suggest that MAG-based analyses have unique advantages over relying solely on unassembled metagenomic reads, culturing fecal indictor organisms or 16S rRNA amplicon sequencing. Members of the identified genus are unlikely candidates for culture-based studies investigating animal waste contamination as this role is dominated by more readily cultivated fecal indicator organisms such as *Escherichia coli* (Lau and Ingham, 2001; Rufete et al., 2006; Anjum et al., 2021). However, analysis of our slurry metagenomes showed that reads assigned to *Proteiniphilum* sp. were between 15- and 41-times more prevalent than those classified as *Escherichia* sp., indicating that while they lack the clinical status of *E. coli* as a potential pathogen, they are more representative of the cattle slurry microbiome and therefore more suitable indictors of contamination. *Proteiniphilum* spp. are hydrolytic facultative anaerobes, first isolated from anaerobic sludge (Chen and Dong, 2005; Hahnke et al., 2016). The genus is known to be abundant in cattle waste (Pandey et al., 2018), has been isolated from cattle rumen (Kim et al., 2020), and is prevalent in mesophilic digestate produced from cattle waste (Hahnke et al., 2016; Garbini et al., 2022). Furthermore, although 16S rRNA amplicon datasets can enable inference of bacterial taxa transmitted from slurry to soil, comparing MAGs generated from slurry (source) and field soil (sink) allows more robust investigation into whether the populations of a given taxon are likely to be closely related at the genomic level.

It is important to acknowledge, however, that MAGs can often represent an aggregate of closely related genomes, rather than a discrete genome (Setubal, 2021). It is therefore important to interpret their significance with care. Application of long-read or hybrid sequencing, together with improved assembly and binning methods may minimize this issue in time. Nonetheless, we suggest the recovery of these MAGs provides sufficient evidence of bacterial transfer which goes beyond differential abundance testing based on unassembled reads. This is because these MAGs recruited many more reads in the source than the sink (Figures 6A,B) and, critically, are genetically ‘conserved’ in both. In addition, read recruitment in the control field soil remained low in 2018, meanwhile, over the same period, their relative abundance increased in the treated site following slurry application in May 2018 (Figure 6B). Additionally, we show that there is good breadth of coverage of bin 127 in treated soil immediately after slurry application and in May 2018, whereas coverage of bin 127 was poor across both January and May 2018 in untreated soil samples (Supplementary File 4). We do not believe this MAG is an artifact of the binning process since it is unlikely that such similar MAGs would have arisen independently in both soil and slurry metagenomes; environments which are clearly distinct from one another.

To investigate the introduction and persistence of this slurry-associated biomarker, we used the *Proteiniphilum* slurry MAG (bin 127) to estimate relative abundance over time in the slurry (Figure 6A), treated soil, and untreated soil samples (Figure 6A). We observed an increase in the abundance of the *Proteiniphilum* MAG immediately after the first 2017 slurry application, however the bacterial genome-normalized abundance declined to pre-application levels within 56 days. Enrichment of *Proteiniphilum*-associated 16S rRNA amplicons in bulk soil following swine manure-amendment has been reported (Wolters et al., 2018). Wolters et al. (2018) found that the relative abundance of *Proteiniphilum* returned to pre-application levels within 156 days. However, the study did not incorporate multiple fertilization events and did not collect further soil samples during the intervening period; it is therefore possible that *Proteiniphilum* had declined much earlier than 156 days. Likewise, Begum et al. (2024) found that a single swine slurry application produced a transient increase in *Proteinipilum* in soil using 16S rRNA amplicon data. We show subsequent slurry application events in 2017 did not appear to coincide with further increases in the abundance of the slurry-associated *Proteiniphilum* MAG. Despite successive slurry applications, the relative abundance of bin 127 declined and then remained relatively stable in soil throughout the remainder of the year (Figure 6B). At the beginning and end of the closed period, the estimated abundance closely resembled the distribution in soil prior to the first application of the season.

A possible explanation for the absence of a progressive increase in *Proteiniphilum* with repeated slurry applications could be that input declined. *Proteiniphilum* abundance showed a contemporaneous decline in the slurry tank (Figure 6A). Although previously reported analysis of the slurry tank taxonomy shows the stability of dominant phyla over time (Baker et al., 2022), this does not extend to lower taxonomic ranks. This suggests, that even though the slurry tank continues to receive fresh material throughout the year, environmental factors and farm practice may influence the relative abundance of slurry tank bacterial taxa. Alternatively, environmental factors affecting the field soil may be responsible for the declining persistence of slurry borne *Proteiniphilum*. For example, Sun et al. (2022) demonstrated that the prevalence of *Proteiniphilum* was positively associated with organic acids which increased following cattle amendments applied to field soil in spring and early summer but not late summer. We show that read recruitment of the *Proteiniphilum* MAG increased from January to May 2018 in the slurry-treated site, while it remained stable over the same period in the untreated site (Figure 3B). Given the similar composition and location of the two field sites, the divergent recruitment of reads suggests that the addition of slurry rather than the local environment was responsible for the observed increase in the slurry-treated site.

Therefore, while both *Proteiniphilum* and macrolide-lincosamide-streptogramin (MLS) resistance genes were associated with the slurry source, and exhibited transient enrichment following slurry application, their temporal dynamics were not linked directly. The relative abundance of the *Proteiniphilum* MAG increased immediately after slurry application and returned to baseline within approximately 8 weeks, whereas MLS ARGs displayed enrichment patterns that varied seasonally and were sometimes evident even when *Proteiniphilum* abundance had already declined. Furthermore, no ARGs were detected within the *Proteiniphilum* MAG itself, suggesting that the transient increase in this bacterium was not a direct vector for MLS gene dissemination. These observations imply that bacterial transfer and ARG transfer can occur as partly independent processes: while slurry-borne bacteria such as *Proteiniphilum* may transiently establish in soil, ARG enrichment may also result from horizontal gene transfer among native soil taxa or from selective pressures acting on the existing resistome. This distinction highlights the complexity of slurry-soil interactions and underscores that changes in ARG abundance cannot be assumed to mirror the persistence of specific slurry-borne organisms.

Although only a limited number of high-quality metagenome-assembled genomes (MAGs) were recovered, these genomes provide valuable insight into the fate of specific slurry-borne microorganisms within the soil environment. The *Proteiniphilum* MAG, for example, illustrates the transient establishment and subsequent decline of a slurry-associated bacterium following application. However, these MAG-based observations are used here as representative case studies rather than as the sole basis for community-wide interpretation. Broader inferences about soil microbial and resistome dynamics are supported by compositional and diversity analyses derived from the complete metagenomic dataset. Together, these complementary lines of evidence provide confidence that the observed temporal changes reflect true community-level responses, even though individual genome reconstructions remain limited.

It is important to note that the slurry-treated and untreated field sites differed slightly in soil texture (heavy clay loam versus silty clay loam). Variations in clay and silt content can influence soil moisture retention, aeration, and organic matter dynamics, thereby shaping microbial community composition and the persistence of extracellular DNA or antibiotic resistance genes (e.g., Carson et al., 2010). Such physicochemical differences may therefore contribute to some of the baseline variation observed between the two field sites. However, both fields are geographically proximate, share the same climatic conditions, and have been maintained as permanent grassland for many years. Moreover, the key temporal patterns observed in this study (such as the transient enrichment of macrolide-lincosamide-streptogramin (MLS) resistance genes following slurry application and the short-term detection of a slurry-associated *Proteiniphilum* MAG) were closely linked to the periodic timing of slurry inputs rather than static site characteristics. These findings suggest that while soil texture may partly modulate microbial and resistome profiles, it is unlikely to account for the principal treatment-related effects observed here.

## 5 Conclusion

This in-situ field study illustrates the complexity of the farming environment and that applying a rigid approach to managing AMR on farms is unlikely to yield consistent results. We demonstrate that at the studied dairy farm, the soil resistome was primarily enriched in indigenous ARGs rather than those associated with cattle slurry. We also show that the abundance and diversity of dominant ARGs in slurry varies over time, suggesting that more attention should be paid to whether the timing of amendments influences the potential persistence of slurry ARGs (particularly MLS resistance genes) and bacteria (as demonstrated by the *Proteiniphilum* MAG) in field soil. Future studies investigating the dispersal of AMR in agricultural settings should therefore aim to sample across seasons to reflect the temporal heterogeneity of soil and animal waste amendments. Furthermore, obtaining deeply sequenced metagenomes of agricultural soil before and after slurry application provides opportunities to examine the transfer and persistence of animal waste-borne bacteria at the genomic level. Our study highlights that understudied organisms may constitute the majority of organisms transferred to soil from cattle slurry, and non-culture-based techniques may be required to quantify their survival.

While previous work on the same farm has shown that storage methods could help control AMR in slurry tanks, the present work suggests that slurry application time may also be important for minimizing the transfer of AMR to field soils. Further research is required to understand if specific generalized guidelines are feasible.

## Data Availability

Additional data can be found in the Supplementary material. Metagenomic sequences were uploaded to the European Nucleotide Archive (ENA) under the following accession: PRJEB38990. Individual accessions and descriptions of samples analysed in this study are detailed in Supplementary File 2.
