# Supplementary material for: Temporal signals in dairy cattle slurry and fertilized field soil resistomes and bacterial communities
Source: Front Microbiol. 2026 Jan 20;16:1666851. doi: 10.3389/fmicb.2025.1666851 (PMC12865810; doi:10.3389/fmicb.2025.1666851)
Supplement: Supplementary file 1 [file Data_Sheet_1.ZIP › Williams et al. Supplementary Material/Supplementary Graphs and Tables.docx]

**Supplementary Figures**


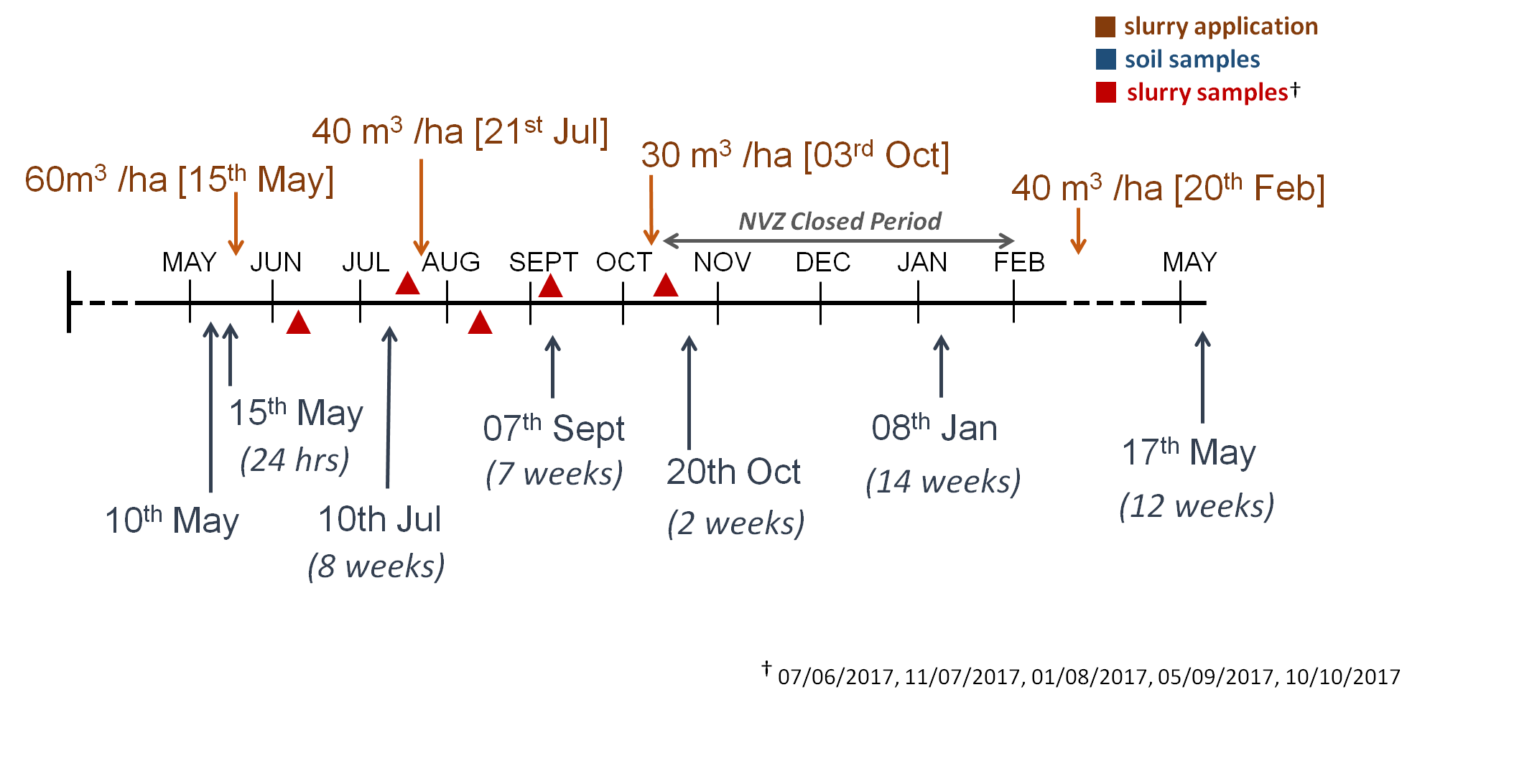


**Supplementary Figure 1.** Experimental timeline detailing soil and slurry collection, in addition to slurry application events. Arrows above the timeline denote slurry application events; application rate and date of application are shown. Arrows below the timeline indicate soil sample collection dates; time since the last slurry application is shown in brackets. Triangles identify slurry sample collection dates, see † for exact dates.


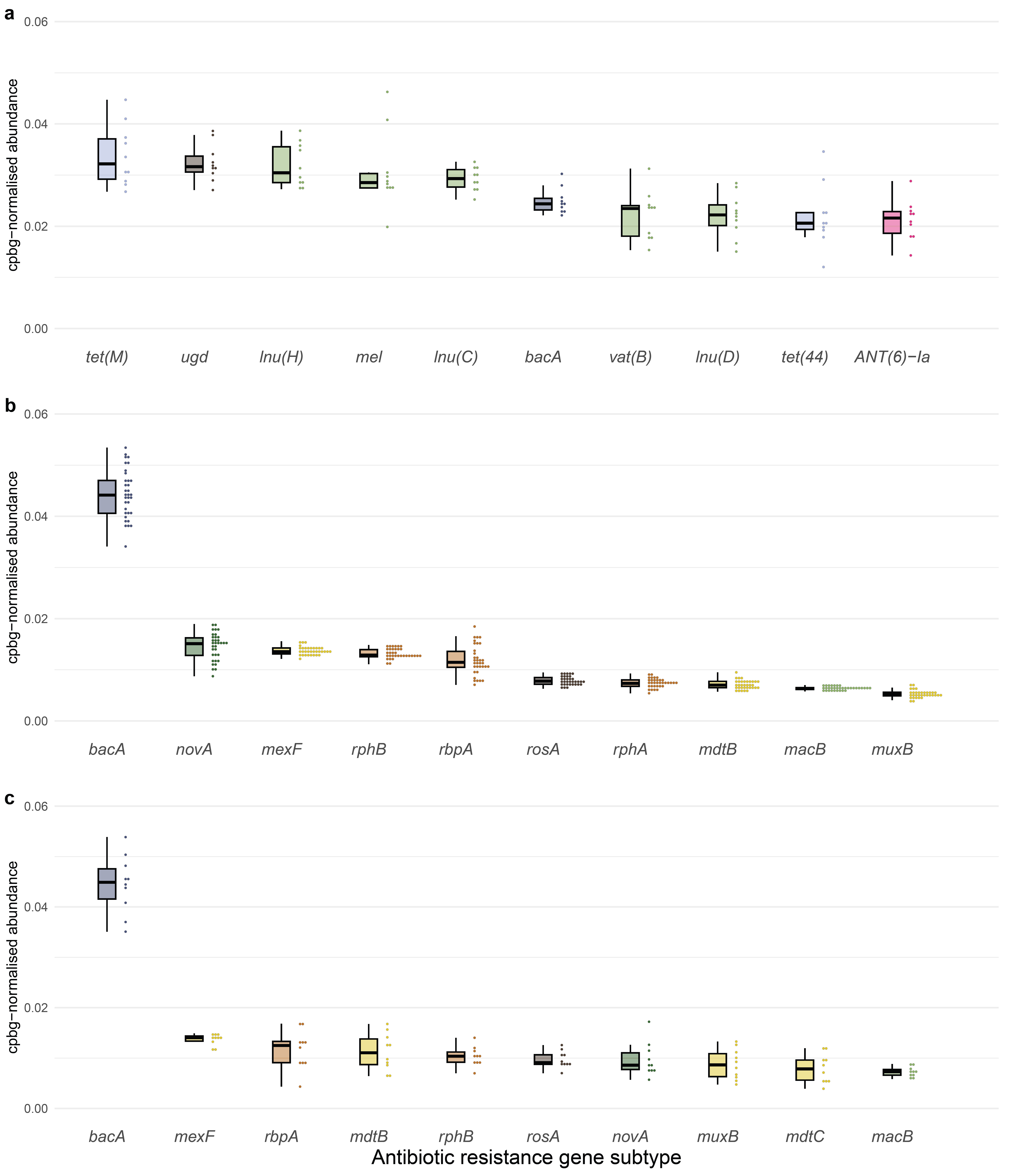


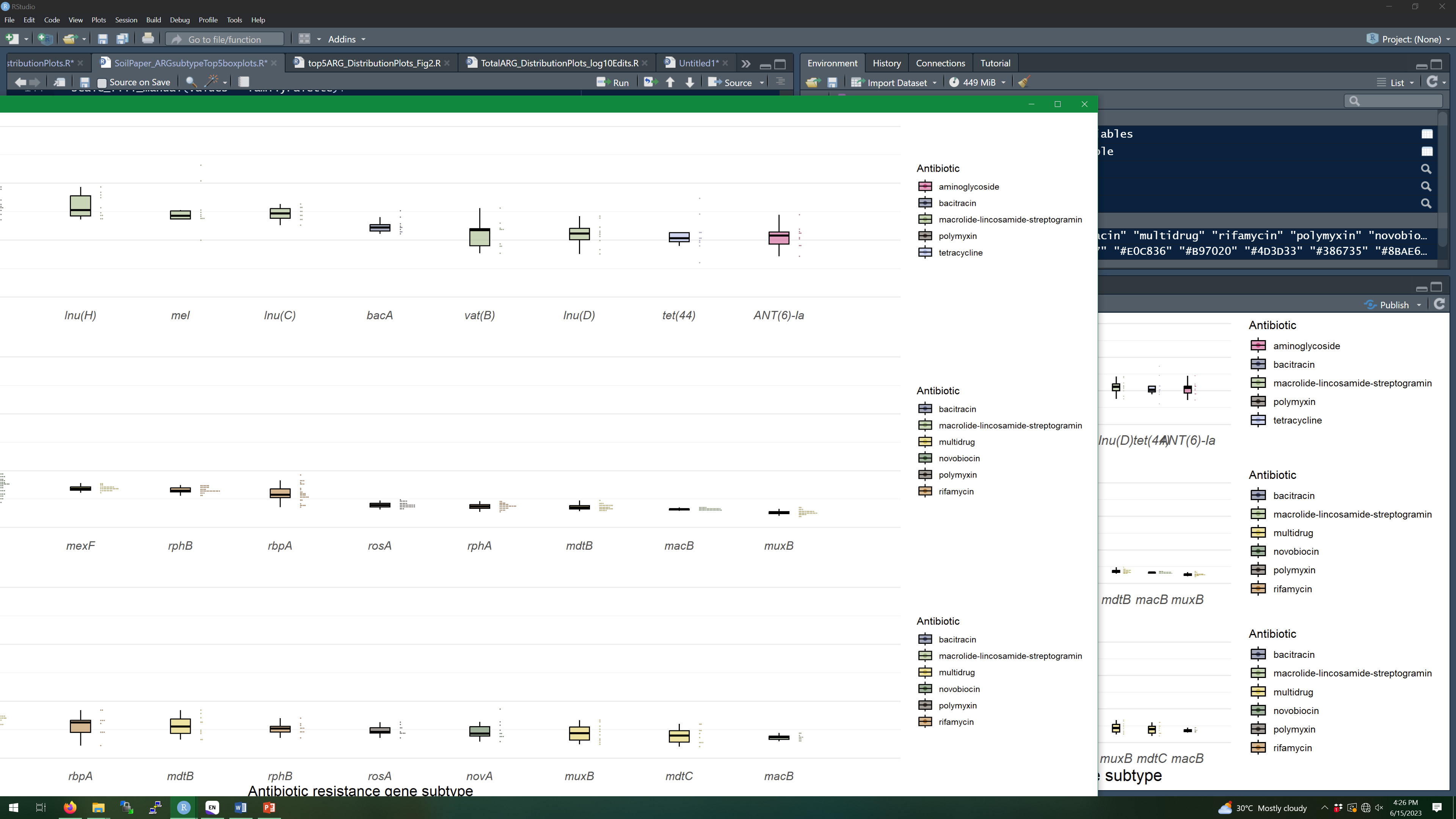

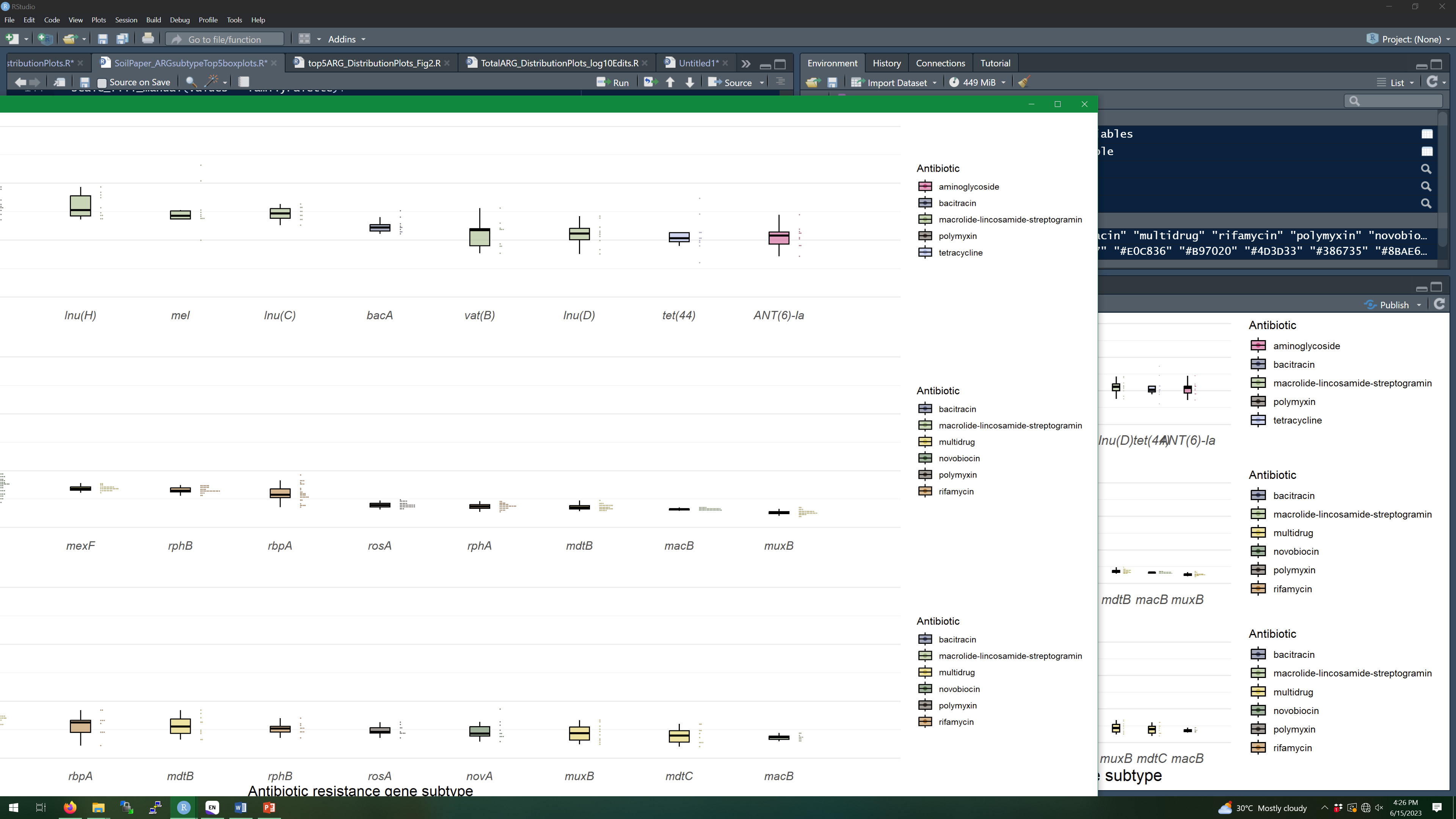

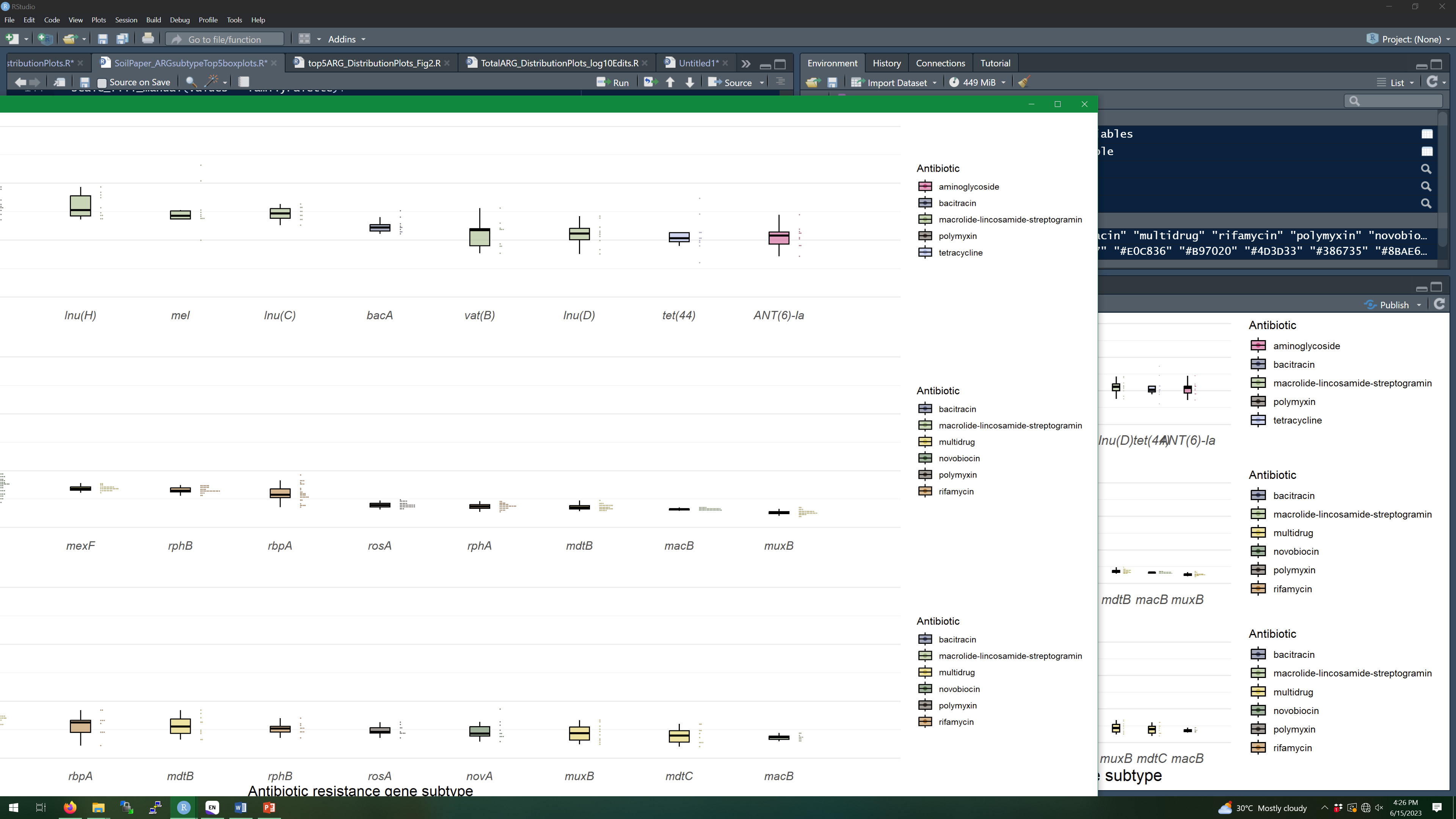


**Supplementary Figure 2.** Boxplots with accompanying dot-plots showing 10 most abundant antibiotic resistance gene subtypes in the screened environments according to mean copies per bacterial genome (cpbg) -normalised abundance: (**a)** slurry (*n* = 10); (**b)** slurry-impacted site (*n* = 34) and (**c)** soil untreated site soil (*n* = 10). Colour denotes antibiotic category (see key).


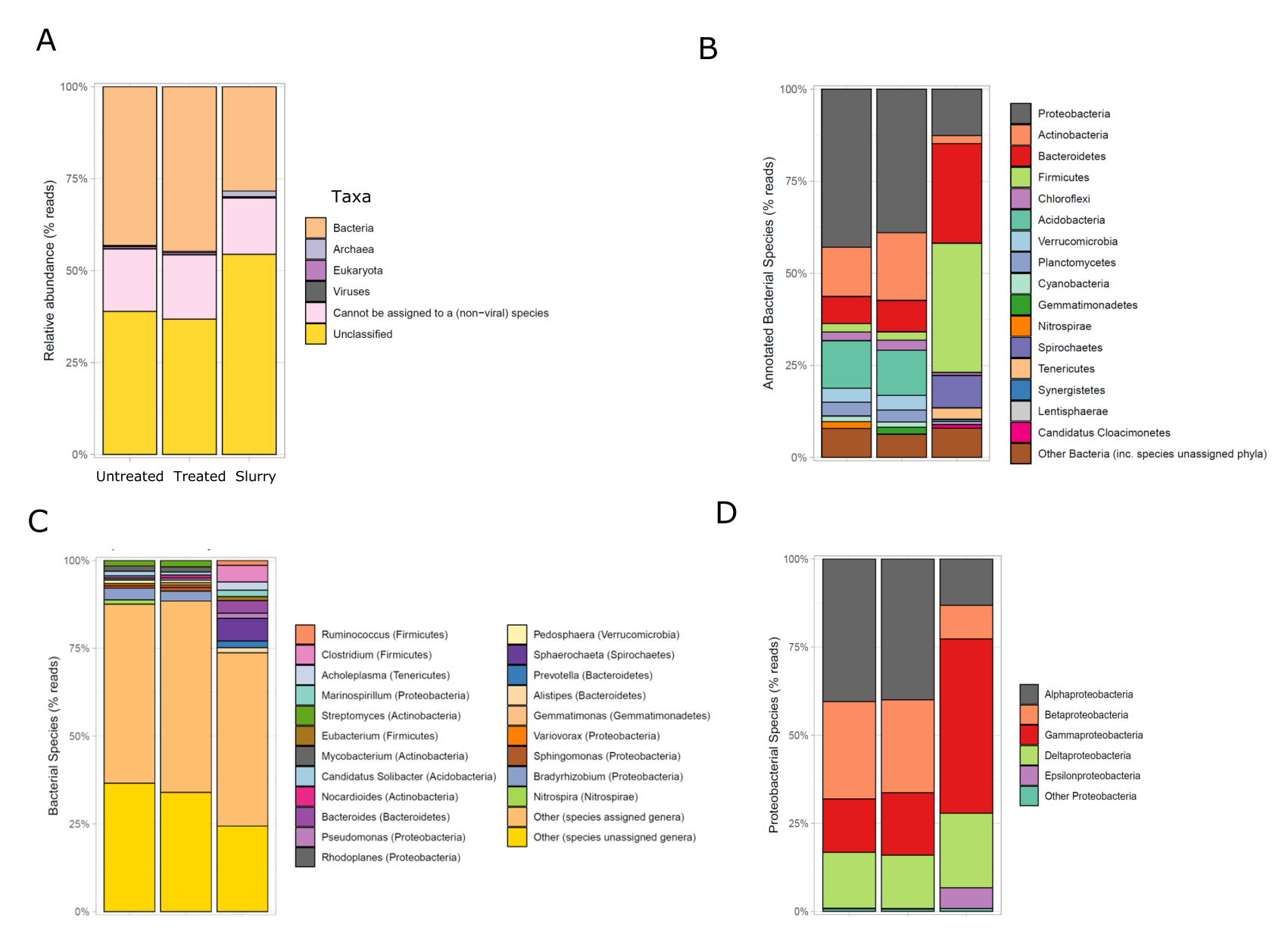


**Supplementary Figure 3.** Stacked bar charts detailing average taxonomic composition of reads. Untreated refers to field site soil with no history of slurry application, treated refers slurry-impacted site soil and slurry refers to slurry from the slurry tank. (A) Breakdown of all reads by domain, including those unclassified by Kaiju. (B) Ten most abundant phyla associated with bacterial reads assigned to species level. (C) Ten most abundant genera associated with bacterial reads assigned to species level. (D) Summary of Proteobacterial class based on reads assigned to Proteobacterial species.


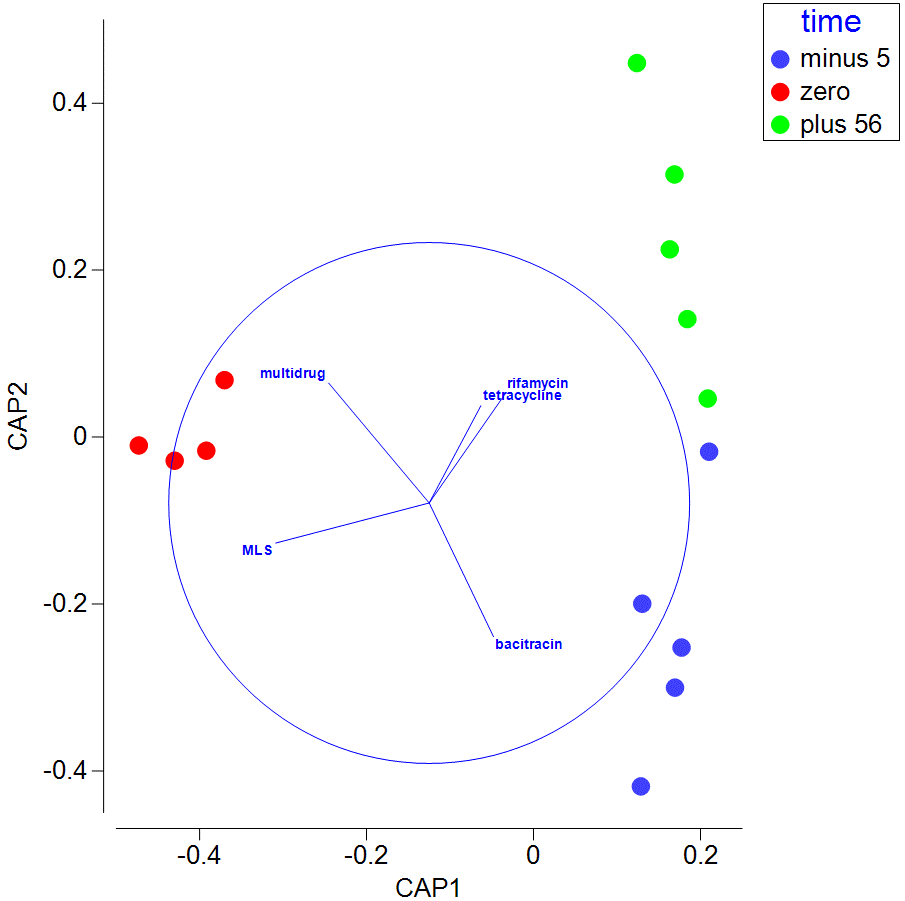


**Supplementary Figure 4.** Discriminant CAP of sample resistome based upon bacterial genome- normalised ARG abundance (Hellinger distance) leading up to and after the first slurry application of 2017 at the treated site. First squared canonical correlation ($\delta_{1}^{2}$) = 0.9851, $\delta_{2}^{2}$ = 0.7474. Sum of canonical eigenvalues = 1.73, *p*_perm_ = 0.002. Mis-classification error of a leave-one-out cross validation = 21.4%. Vector overlays represent multiple partial correlations (*r* > 0.4) of the abundance of individual antibiotic resistance gene categories and CAP axis scores. The length and direction of each vector indicates the strength and sign, respectively, of the relationship between that variable and the CAP axes. The circle is a unit circle (radius = 1.0), whose relative size and position of origin is arbitrary with respect to the underlying plot.


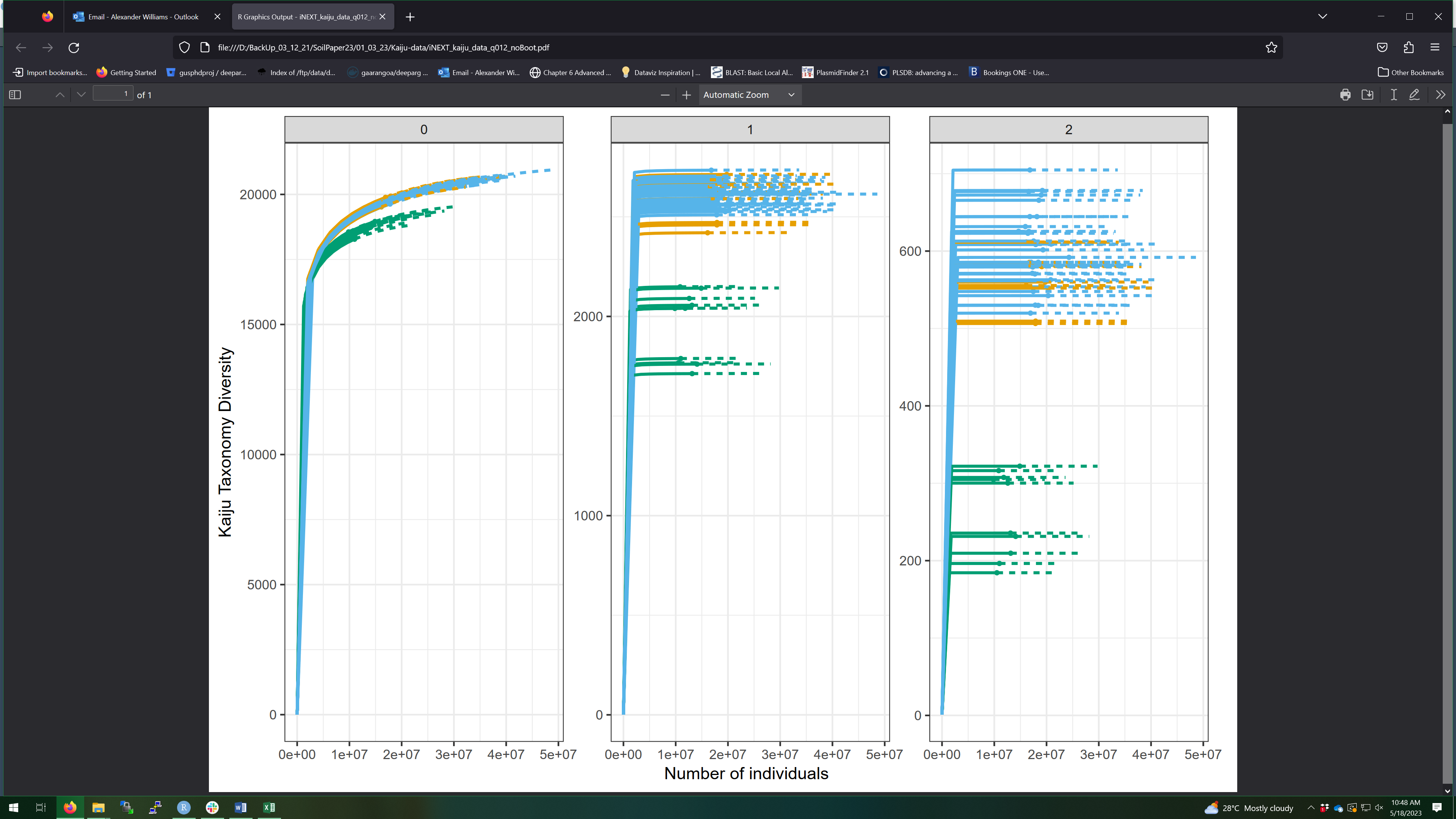


**Supplementary Figure 5.** iNEXT diversity estimates for bacterial taxa, showing the greater diversity of soil relative to slurry. Colour denotes sample source as follows: slurry (green), treated site soil (blue) and untreated site soil (orange). Solid line denotes observed values, dashed line denotes extrapolations.


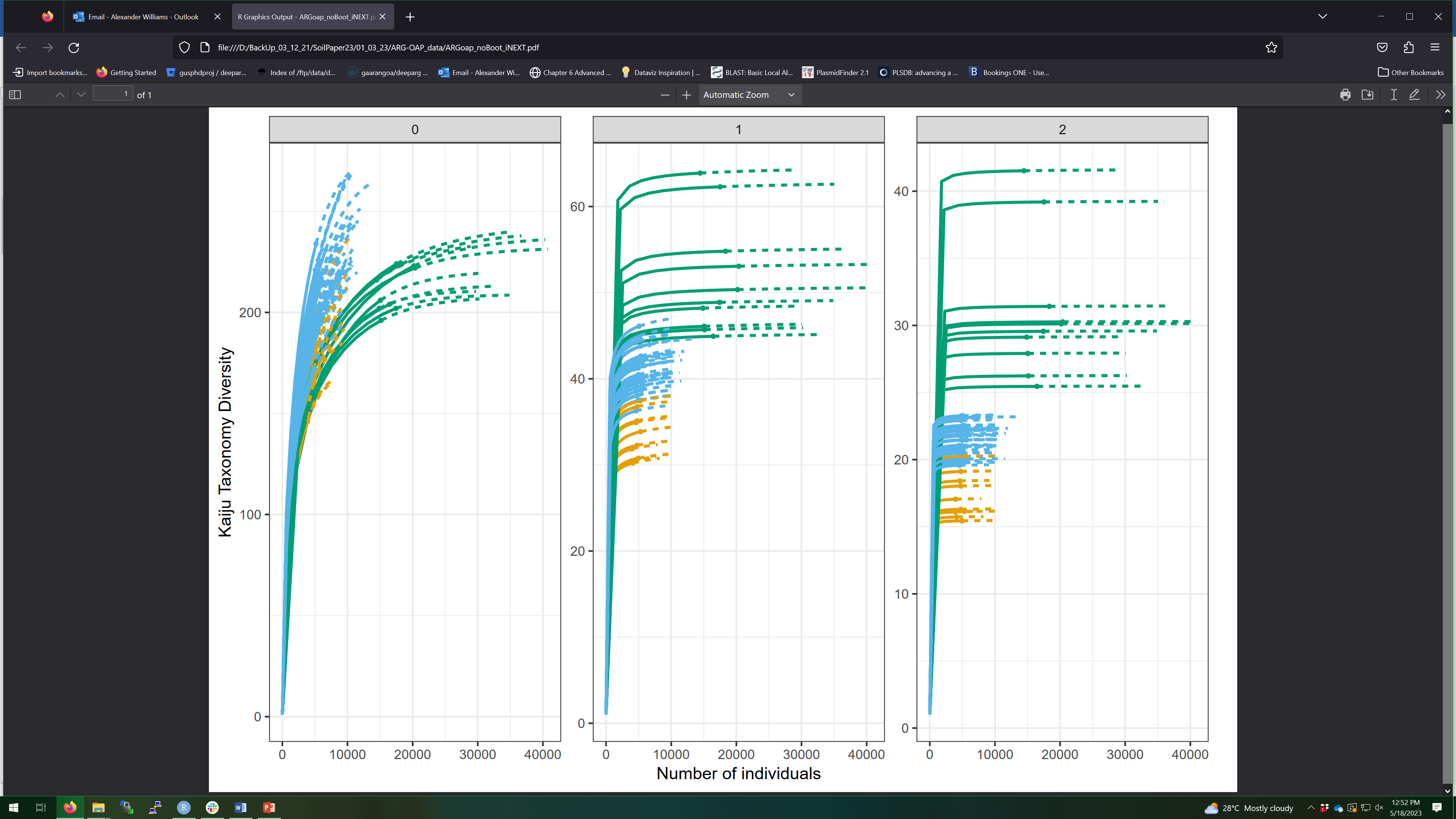


**Supplementary Figure 6.** iNEXT diversity estimates for antibiotic resistance genes (ARGs) detected by ARG-OAP, showing the greater diversity of soil relative to slurry in q0 (richness), while iNEXT shows that the slurry has a greater diversity when reviewing dominant ARGs (q2). Colour denotes sample source as follows: slurry (green), treated site soil (blue) and untreated site soil (orange). Solid line denotes observed values, dashed line denotes extrapolations.

**
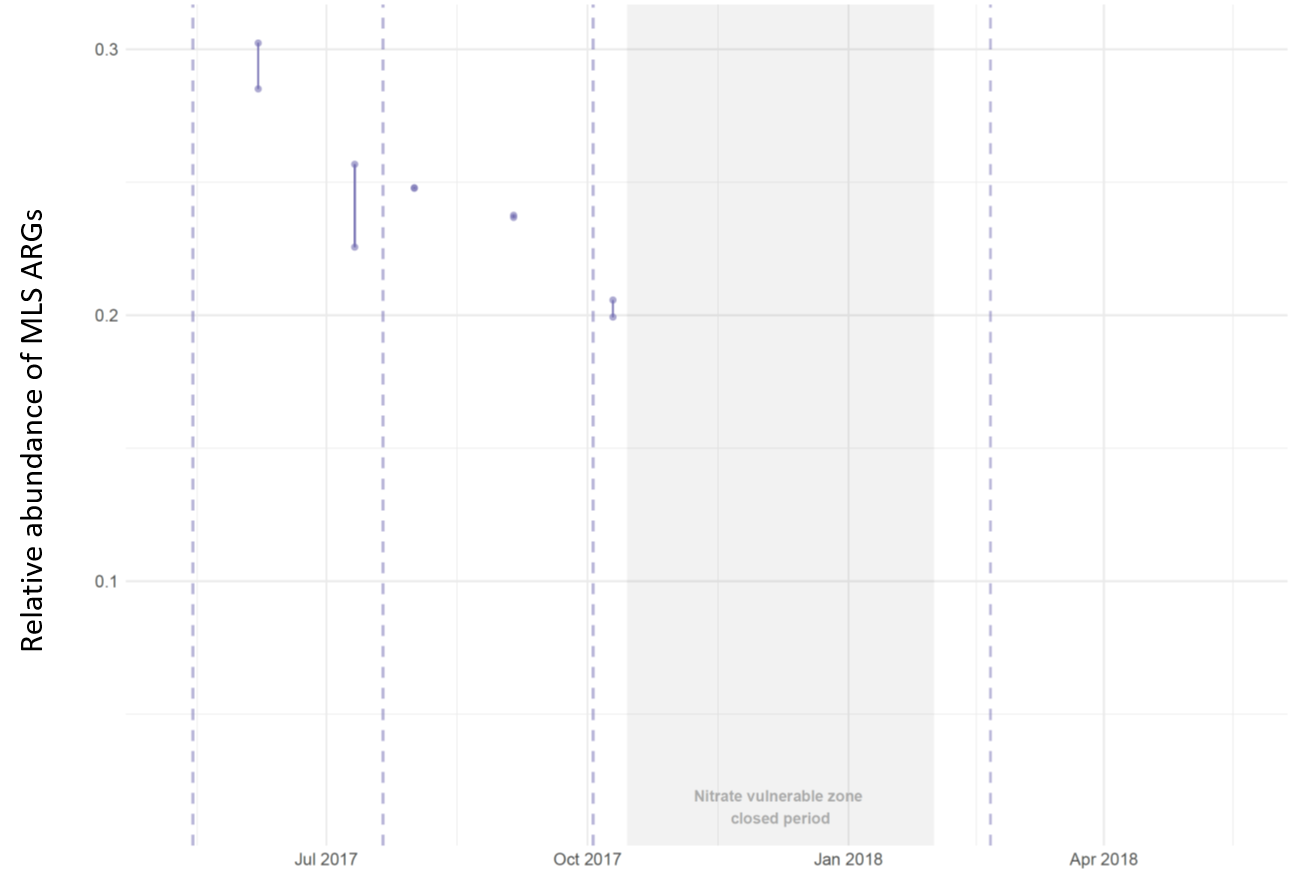
**

**Supplementary Figure 7.** Relative abundance of MLS ARGs (copies per bacterial genome) showing a trend in their decline in the slurry tank from June to October 2017. Vertical dashed lines (purple), indicate slurry application events. Range bars are displayed.

**Supplementary Tables**

**Supplementary Table 1.** Pairwise testing of bacterial phyla compositions.

**Supplementary Table 2.** Pairwise contrasts of *Proteiniphilum* sp. bin 127 bacterial genome- normalised abundance in January and May 2018 in the treated and untreated site soils. Note that the treated site received an application of slurry in February 2018. Neither site received slurry between October or January.

| **Contrast** | **Estimate** | **SE** | **df** | ***t* ratio** | ***p*_adjust_** |
| --- | --- | --- | --- | --- | --- |
| untreated, Jan : untreated, May | 2.6 | 2.27 | 16 | 1.147 | 0.2683 |
| untreated, Jan : treated, Jan | -3.2 | 2.27 | 16 | -1.41 | 0.2127 |
| untreated, Jan : treated, May | -10.2 | 2.27 | 16 | -4.5 | 0.0011 |
| untreated, May : treated, Jan | -5.8 | 2.27 | 16 | -2.56 | 0.0316 |
| untreated, May : treated, May | -12.8 | 2.27 | 16 | -5.65 | 0.0002 |
| treated, Jan : treated, May | -7 | 2.27 | 16 | -3.09 | 0.0141 |

| **Sample type** | **t** | ***p*_perm (adjust)_** | **unique perm.** | |  |
| --- | --- | --- | --- | --- | --- |
| slurry: slurry-treated site | 61.361 | 1x10^-5^ | 81701 |  | |
| slurry: untreated site | 29.859 | 1x10^-5^ | 82087 |  | |
| slurry-treated site: untreated site | 6.4625 | 1x10^-5^ | 94709 |  | |
|  |  |  |  |  | |
| **Days after first slurry application of May 2017** |  |  |  |  | |
| 5 days before: <24 hours | 1.9577 | 0.0483 | 126 |  | |
| 5 days before: 56 days | 1.6528 | 0.0483 | 126 |  | |
| <24 hours: 56 days | 3.5746 | 0.0231 | 126 |  | |

**Supplementary Excel Files**

**Supplementary File 1.** The key physiochemical properties (averages) for the soil and slurry samples included in the present study.

**Supplementary File 2.** Detailed description of accessions associated with all soil and slurry metagenomes analysed in this study.

**Supplementary File 3.** Description of MAGs recovered from slurry and soil samples examined in this study.

**Supplementary File 4.** Coverage of bin 127 of individual contigs in each sample analysed in the present study.
